# Thermodynamic Criterion for Quasi-Static Tearing of Elastic and Elastic–Plastic Fracture Specimens, and Its Validation from the Empirical Structure of Load–Displacement Diagrams

**DOI:** 10.3390/ma19204340

**Published:** 2026-10-15

**Authors:** Victor A. Snyder

**Affiliations:** Soil Physics Laboratory, Department of Agroenvironmental Sciences, University of Puerto Rico Agricultural Experiment Station, 1193 Guayacan Street, South Botanic Gardens, San Juan, PR 00926, USA; victor.snyder@upr.edu; Tel.: +1-(787)-949-9661

**Keywords:** elastic–plastic fracture criteria, fracture thermodynamics, Griffith and Irwin criteria, fracture stability, energy dissipation rate, J-integral

## Abstract

A global thermodynamic criterion is formulated for quasi-static crack extension in elastic and elastic-plastic fracture specimens. It accounts for crack extension accompanied by both large-scale plastic deformation and elastic unloading, which are excluded in LEFM and J-integral theories. Underlying assumptions are the first and second laws of thermodynamics, and an internal energy function where the independent variables are load-point displacement, entropy and crack extension, with the latter assuming the role of internal state variable. The criterion takes the form I = D, where I is an elastic-plastic crack driving force and D is the energy dissipation rate, identified thermodynamically as the sum of heat dissipated per unit of crack extension plus the rate of change of a residual Helmholtz energy parameter admitting non-dissipative irreversible energy forms such as residual elastic energy and Griffith surface energy. The thermodynamic criterion is validated by comparison with energy relations implicit in the structure of experimental load–displacement diagrams. For elastic–plastic specimens exhibiting linear elastic unloading behavior, a simple relation arises between thermodynamic driving and resistance forces and an excess elastic stress field ahead of growing cracks. Instability criteria are formulated for fixed grips and fixed load conditions. The original Griffith and Irwin fracture criteria are derived from the thermodynamic criterion.

## 1. Introduction

This paper re-examines the problem of applying classical macroscopic thermodynamic methods to the modeling of quasi-static crack extension in elastic and elastic–plastic fracture specimens.

The problem was first considered in rudimentary form by Griffith [1] in his famous energy criterion for brittle fracture. This criterion, adapted by Orowan [2,3,4] to distinguish between fixed-grips and fixed-load conditions, may be written for a linear elastic plate specimen of unit thickness and crack length *a* subjected to an external load P and load-point displacement X, as

(1) $$-{\left(\frac{\partial U_{e}}{\partial a}\right)}_{X}da={\left(\frac{\partial \Pi_{e}}{\partial a}\right)}_{P}da=2\gamma \;da$$

where $U_{e}$ is the (global) elastic energy of the specimen, $\Pi_{e}\equiv PX-U_{e}$ is the complementary energy and γ is the specific crack surface energy. In 1948, Irwin [5] postulated that Griffith’s criterion could be extended to elastic–plastic conditions by adding a plastic dissipation term to the surface tension parameter. This effectively modified (1) to

(2) $$-{\left(\frac{\partial U_{e}}{\partial a}\right)}_{X}da={\left(\frac{\partial \Pi_{e}}{\partial a}\right)}_{P}da=2\gamma da+dU_{p}\;\approx dU_{p}$$

where $U_{p}$ is the energy of plastic deformation in material surrounding the crack, and the final identity on the right arises from Irwin’s approximation $dU_{p}\gg 2\gamma da$.

Although (1) and (2) were foundational in fracture mechanics, their use has been limited. The application of (1) is restricted by the experimental indeterminacy of the surface tension parameter γ and the fact that the criterion admits only small-scale plasticity. The use of (2) under ductile conditions is limited by the indeterminacy not only of γ but also of the elastic parameters $U_{e}$ and $\Pi_{e}$, the latter arising from indeterminate amounts of residual or “cold working” elastic energy accumulated during cyclic loading processes involving spatially non-uniform plastic deformation [6,7,8]. Added to this is the collapse of the unique LEFM relation between driving force derivatives and crack–tip stress distribution under ductile conditions, considered an inconvenience compared to the LEFM case where a single parameter accounts for both stress distribution and energy relations associated with fracture [9,10]. Such limitations led to an elastic–plastic adaptation of the J-integral [9,10,11,12,13,14,15], still widely used as a macroscopic tearing criterion.

J-integral theory [9,10,11,12,13,14,15] was originally formulated for non-linear elastic conditions, in which case ***J*** is formally and physically equivalent to the driving force partial derivatives ${\left(\partial U_{e}/\partial a\right)}_{X}\;and\;{\left(\partial \Pi_{e}/\partial a\right)}_{P}$ in (1). Adaptation to the elastic–plastic domain was achieved by formally treating plastic deformation prior to incipient fracture as non-linear elastic. The result was a “pseudo-elastic” parameter preserving the same partial derivative form and contour-invariance as the elastic **J** integral, although losing its thermodynamic meaning as an elastic (Helmholtz) energy release rate. The thermodynamic inconsistency is partly offset whenever ***J*** contour measurements in small fully plastic specimens can be reliably extrapolated to far-field elastic contours in large specimens, since in the latter case the elastic interpretation of ***J*** is recovered [14,15]. However, for ***J*** measurements restricted to the fully plastic domain, indistinction between elastic and plastic deformation remains thermodynamically problematic. A redeeming feature in this case is the unique HRR relation between ***J*** and the crack tip stress field for proportional loading up to incipient tearing [16,17], allowing the use of ***J*** as a characteristic stress intensity parameter at fracture initiation or at least for small or moderate crack extension where unloading effects are ignorable. But difficulties remain if one desires to establish a thermodynamically meaningful condition on ductile fracture, particularly when crack extension is accompanied by significant amounts of both plastic deformation and elastic unloading.

It is precisely within this context that Griffith and Irwin criteria (1) and (2) merit renewed attention. Particularly notable is their formal and physical similarity to models of irreversible processes that would appear much later within the framework of thermodynamics with internal variables (T.I.V.). The similarity may be seen by comparing fracture criteria (1) and (2) to Rice’s well known T.I.V. constraint on inelastic constitutive laws [18], which in Gibbs’ time-implicit notation [19] may be written for fixed-grips conditions as

(3) $$-{\left(\frac{\partial A}{\partial \varepsilon }\right)}_{X}d\varepsilon =dq_{diss}\geq 0$$

Here, A is the Helmholtz energy, X and P are characteristic strains and stresses, respectively, and $\;dq_{diss}$ is the dissipated heat. The parameter ε is an internal state variable, defined generally as some measurable but not directly controllable property of the system that allows for its history dependence to be accounted for [20,21]. The first point of similarity to be noted is that variations in elastic energy $U_{e}$ in (1) and (2) are essentially equivalent to the changes in Helmholtz energy A in (3). Secondly, the plastic deformation energy $dU_{p}$ of (2) is synonymous with the dissipated heat $dq_{dis}$ of (3). Thirdly, the crack length parameter *a*, which is a ***measurable but not directly controllable property of fracturing bodies***, assumes the same formal role in (1) and (2) as does the internal state variable ε in (3). Finally, and perhaps most significantly, all three formulations, (1), (2) and (3), represent irreversible material processes driven by elastic unloading. The only major difference, apart from the specific types of irreversible processes considered, is one of scale. That is, fracture criteria (1) and (2) are global constraints whereas (3) is formulated at the scale of a material continuum element. The above similarities are rather remarkable considering that, according to Maugin’s historical accounts [20,21], the formal T.I.V concepts underlying (3) would not be in place until decades after fracture criteria (1) and (2) were formulated.

One of the first analyses of fracture within a formal T.I.V. framework appeared in a 1978 paper by Rice [22]. The parting assumptions were the first and second laws of thermodynamics and existence of a (global) Helmholtz energy function for a fracturing body of form A(X,a,T), where load-point displacement X and temperature T were the controllable independent parameters and crack length *a* assumed the role of internal state variable. For isothermal conditions, this led to a fixed-grips condition on quasi-static crack extension, which in time implicit notation can be written as

(4) $$-{\left(\frac{\partial A}{\partial a}\right)}_{X}da=dq_{dis}\geq 0$$

where formal similarity with (3) is evident, with the distinction that (3) is a local criterion whereas (4) is global. Criterion (4) is arguably applicable to both elastic and elastic–plastic tearing conditions. However, Rice was interested in applying it to the special problem of Griffith cracks. This required relating the Helmholtz energy A to parameters specific to the Griffith model, which Rice achieved through the constitutive relation

(5) $$A\left(a,X\right)=2\gamma a+U_{e}\left(a,X\right)$$

Substituting constitutive relation (5) into thermodynamic constraint (4) gives

(6) $$-\left[2\gamma +{\left(\frac{\partial U_{e}}{\partial a}\right)}_{X}\right]da=dq_{dis}\geq 0$$

For the non-dissipative condition $dq_{dis}=0$, (6) reduces to

(7) $$-{\left(\frac{\partial U_{e}}{\partial a}\right)}_{X}da=2\gamma \;da$$

which is the fixed-grips version of Griffith’s criterion (1).

Given that T.I.V. theory pertains to irreversible processes, its application to fracture carries the connotation that crack extension is irreversible. In considering this issue, Rice (22) cited certain cases of apparently reversible fracture, but noted that for the most part, irreversibility is the norm. Maugin [21] has been more categorical, classifying fracture generally within the domain of irreversible material behaviors. A similar viewpoint, usually not stated explicitly, underlies much of the theoretical structure of fracture mechanics presented in standard textbooks [9,10]. A case in point is the unloading compliance function $C(a)\equiv {\left(\frac{\partial X}{\partial P}\right)}_{a}$, which is based on the assumption that crack length remains fixed during unloading processes. Irreversibility is also implicit in partial derivatives of form ${\left(\frac{\partial }{\partial a}\right)}_{X}$ and ${\left(\frac{\partial }{\partial a}\right)}_{P}$, which are ubiquitous throughout fracture mechanics. Such derivatives, which imply mutual independence of crack length and load coordinates X or P, would be undefined if crack length varied reversibly with load coordinates during cyclic loading and unloading. In this respect, it is worth noting that the partial derivative representation ${-\left(\frac{\partial U_{e}}{\partial a}\right)}_{X}$ is retained for the crack driving force in Griffith’s criterion (1) even though the thermal dissipation term $dq_{dis}$ is assumed absent. Consistency would then seem to admit some source of irreversibility in addition to thermal dissipation.

Various causes of fracture irreversibility have been postulated in the literature, the most dominant one being thermal dissipation. In brittle elastic materials, dissipation has been attributed to intense heating at the crack tip and associated chemical and structural changes at crack surfaces [21,22]. In ductile fracture, dissipation occurs during plastic deformation of material surrounding the crack tip. However, as anticipated above, non-dissipative sources of irreversibility can also be identified. A documented example is the fracture dislocation glide effect [22,23], whereby irregular surfaces formed on opposite sides of a crack are laterally displaced relative to each other, and therefore cannot fit back together when the system is unloaded. In such systems, even though Griffith surface tension energy may have accumulated during crack extension and exerts a crack closure force during unloading, closure is prevented by the interfering effects of dislocation. A similar situation occurs in the case of residual elastic energy trapped in localized zones within a cracked body due to the “warping” effect of non-uniform plastic deformation fields [6,7,8]. Although residual elastic energy accumulation is, in principle, reversible at a continuum element scale, globally, it is manifested as an irreversible deficit in work exchange between a body and its environment during cyclic loading, a deficit that cannot be attributed to dissipation alone.

As in other areas of thermo-mechanics, the modern trend in fracture thermodynamics has been to reduce the space scale of analysis to the level of a material continuum element [24,25,26]. Extension to the global behavior of a structure is then achieved by volume integration of localized properties and processes. This approach has the obvious advantage of allowing the effects of localized inhomogeneities within a structure on its global behavior to be estimated, ***provided*** sufficiently detailed knowledge of the inhomogeneities and associated micro-processes is available. However, under the extreme gradients and highly disorganized material conditions often encountered near crack tips, well-posed formulations of microstates and microprocesses remain challenging even with advanced mathematical and computational capabilities. This still leaves room for older macroscopic “detail-blind” approaches to fracture thermodynamics, as embodied in Rice’s criterion (4), where system parameters are from the outset defined at a global or “whole body” scale.

In Rice’s global T.I.V. formulation of fracture described above, imposition of constitutive constraint (5) on thermodynamic criterion (4) restricted analysis to the specific case of Griffith cracks. This begs the question of if and how (4) could be extended to elastic-plastic fracture if model-specific constitutive relation (5) was removed and replaced by a more general relation. Simple and dated as such a question may seem, it is an important one, which, to this author’s knowledge, has never been rigorously addressed. Providing an answer to the question is the objective of the present paper.

The primary objective is to derive an elastic–plastic generalization of Rice’s global thermodynamic tearing criterion (4) parting from the first and second laws of thermodynamics and an assumed state function of form U(∄, X, S, *a*), where U is the internal energy of a fracturing body, S is its entropy, X is a characteristic load-point displacement, crack length *a* is an internal state variable, and ∄ accounts for effects of specific specimen shape and loading configuration. A second objective is to provide experimental verification of the thermodynamic criterion. This is achieved by analysis of the empirical structure of load–displacement diagrams observed in fracture tests, leading to energy relations at imminent crack extension that turn out to be equivalent to those arising in the thermodynamic analysis. A third objective is to explore various applications of the thermodynamic model, including stability criteria and relation of thermodynamic driving and dissipative forces to stress concentration ahead of advancing cracks. The Griffith and Irwin fracture criteria are shown to be derivable from the thermodynamic criterion.

These objectives are addressed below in the following order. Section 2 first reviews the well-known structure of experimental load–displacement diagrams obtained during the fracture testing of elastic and elastic–plastic specimens, and shows that a global energy criterion for tearing is implicit in that structure. In Section 3, a thermodynamically derived tearing criterion is formulated, and is validated by showing its equivalence to the empirical energy criterion obtained in Section 2. Section 4 discusses applications of the thermodynamic criterion including stability considerations. Section 5 discusses limitations of the theory. Section 6 provides a summary and conclusions. The Appendix A includes sample data and calculations used in the stability analysis.

## 2. Derivation of Energy Criterion from the Structure of Load–Displacement Diagrams Obtained in Fracture Tests of Elastic and Elastic–Plastic Specimens

This section examines the structure of experimental load–displacement diagrams obtained in fracture tests of elastic and elastic–plastic specimens, and shows that this structure implies an energy criterion for crack extension. The analysis first focuses on the common case of materials exhibiting linear elastic unloading behavior, characteristic of LEFM specimens and also many elastic–plastic materials such as structural metals for which (linear) unloading compliance methods are routinely used to estimate crack extension [10,27]. For such materials, the relation between load–displacement diagrams and the energy criterion for crack extension can be derived analytically. The analysis then examines elastic–plastic specimens exhibiting non-linear elastic unloading behavior, which can occur in certain materials such as high-strength steels and composites [28]. In such cases, the energy criterion may be derived with reasonable rigor through graphical inspection of load–displacement diagrams.

### 2.1. Analysis of Specimens with Linear Elastic Unloading Behavior

#### 2.1.1. General Structure of Load–Displacement Diagrams

Figure 1 illustrates classic “textbook” load–displacement diagrams for the common case of elastic and elastic-plastic fracture specimens exhibiting linear elastic unloading behavior. The two principal elements in each diagram are a primary “tearing path” (boldfaced line) showing evolution of load coordinates during continuous tearing of the specimen with no intermittent unloading, and a family of elastic unloading paths (dashed lines) arising when primary loading at successive crack lengths is interrupted by cyclic unloading and re-loading events. Loading states on tearing and unloading paths are denoted by primed and unprimed coordinates (X’, P’) and (X, P), respectively. An important property is that each point (X’,P’) on the tearing path is also the upper extremum of an elastic unloading path, with coordinates (X, P) emanating from that point. The slopes of the unloading paths define the inverse of the compliance C. In the case of fully elastic specimens, the unloading curves always intercept the origin (Figure 1a), whereas for elastic–plastic specimens the intercepts with the X-axis are systematically displaced to the right with increasing crack extension (Figure 1b).

#### 2.1.2. Unloading Compliance Function, Elastic Unloading Energy, Complementary Energy and Associated Derivatives

The unloading compliance function $C\left(X,a\right)$ for the linear elastic unloading curve corresponding to crack length a is defined by the following:

(8) $$C\left(X,a\right)=\;\;\frac{\left[X-X_{o}\left(a\right)\right]}{P(X,a)}$$

The independent variable X in (8) is the abscissa of an arbitrary point ***e*** on the elastic unloading path corresponding to crack length *a* (Figure 2). The domain of possible values of X in the compliance function (8) is $X_{o}\left(a\right)\leq X\leq X^{\prime }(a)$,

$X^{\prime }(a)$ is the critical load-point displacement at which the crack begins to extend, and $X_{o}\left(a\right)$ is the “plastic set” or point where the linear unloading curve extrapolates to the *X*-axis. Load P(X,a) is the conjugate of *X* on the unloading path, and is restricted to the domain $0\leq P(X,a)\leq P^{\prime }(a)$, where $P^{\prime }\left(a\right)$ is the critical load required for crack extension. Since the unloading path is linear, the value of $C\left(X,a\right)$ in (8) is independent of the arbitrarily chosen value of X; therefore, ${\left(\partial C/\partial X\right)}_{a}=0$ so that $C\left(X,a\right)=C(a).$ This allows (8) to be written in the functional form

(9) $$C\left(a\right)=\;\frac{\left[X-X_{o}\left(a\right)\right]}{P(X,a)}$$

The apparent elastic energy $U_{ea}(X,a)$, illustrated by area *def* under the unloading curve in Figure 2 is defined by

(10) $$U_{ea}\left(X,a\right)\equiv \int_{X_{o}}^{X}P\left(\tau ,a\right)d\tau =\frac{1}{2}\;P\left(X,a\right)\cdot \;\left[X-X_{o}\left(a\right)\right]$$

where the term “apparent” denotes that $U_{ea}(X,a)$ excludes any indeterminate residual elastic energy existing in the macroscopically unloaded state *P* = 0.

Solving the compliance function (9) for P(X,a) gives

(11) $$P\left(X,a\right)=\frac{\left[X-X_{o}\left(a\right)\right]}{C\left(a\right)}$$

which, substituted into (10), gives $U_{ea}$ in terms of X, $X_{o}\left(a\right)$ and C(*a*) as

(12) $$U_{ea}\left(X,a\right)=\frac{1}{2}{\left[X-X_{o}\left(a\right)\right]}^{2}/C\left(a\right)$$

Taking the partial derivative ${\left(\partial U_{ea}/\partial a\right)}_{X}$ of (12) and again making use of (11) gives

(13) $${-\left(\frac{\partial U_{ea}}{\partial a}\right)}_{X}=\frac{1}{2}{\left[X-X_{o}\left(a\right)\right]}^{2}/{\left[C\left(a\right)\right]}^{2}\cdot \frac{dC\left(a\right)}{da}+\frac{\left[X-X_{o}\left(a\right)\right]}{C\left(a\right)}\cdot \frac{dX_{o}\left(a\right)}{da}$$

(14) $$\;\;\;\;\;\;\;\;\;\;\;\;\;\;\;\;\;\;\;\;\;\;\;\;\;\;\;\;\;\;\;\;=\frac{1}{2}{\;P}^{2}\frac{dC}{da}+P\frac{dX_{o}}{da}\;\;\;\;\;\;\;\;\;\;\;\;\;\;\;\;\;\;\;\;\;(0\leq P\leq P^{\prime }\;)$$

where it should be noted that (14) is defined over the entire loading range $0\leq P\leq P^{\prime }(a)$.

The apparent complementary energy $\Pi_{ea}$, illustrated by area *bcde* in Figure 2, is defined by

(15) $$\Pi_{ea}\equiv PX-U_{ea}=\;PX-\frac{1}{2}\cdot \frac{{\left[X-X_{o}\left(a\right)\right]}^{2}}{C(a)}=PX-\frac{1}{2}\;C(a)\cdot P^{2}$$

where use has been made of (12) and then (11). Solving for X in (11) and substituting the result into (15) yields

(16) $$\Pi_{ea}=\frac{1}{2}\;P^{2}C\left(a\right)+PX_{o}(a)$$

for which the partial derivative ${\left(\partial \Pi_{ea}/\partial a\right)}_{P}$ gives

(17) $${\left(\frac{\partial \Pi_{ea}}{\partial a}\right)}_{P}=\frac{1}{2}{\;P}^{2}\frac{dC}{da}+P\frac{dX_{o}}{da}\;\;\;\;\;\;\;\;\;\;\;\;\;\;\;\;\;\;\;(0\leq P\leq P^{\prime }\;)$$

Comparing (14) and (17) shows that

(18) $${-\left(\frac{\partial U_{ea}}{\partial a}\right)}_{X}={\left(\frac{\partial \Pi_{ea}}{\partial a}\right)}_{P}=\left(\frac{1}{2}{\;P}^{2}\frac{dC}{da}+P\frac{dX_{o}}{da}\right)\;\;\;\;\;\;\;\;\;\;\;\;\;\;\;\;\;\;\;(0\leq P\leq P^{\prime }\;)$$

This indicates that the partial derivative quantities −${\left(\partial U_{ea}/\partial a\right)}_{X}\;and\;\left(\partial \Pi_{ea}/\partial a\right)\backslash_{P}$ are non-linear homogeneous functions of the current load *P* and also equal to each other for a given loading state (X, P). They represent virtual fixed-load and fixed-grips crack driving forces, respectively, i.e., elastic and complementary energy changes that ***would*** occur ***if*** the crack were to extend by an amount d*a* for fixed-grips or fixed load conditions under an arbitrary applied load P. Note that this does not necessarily imply that the crack ***will*** extend under load P. The relation (18) between driving force derivatives and constitutive parameters P, C and $X_{o}$ was pointed out earlier by the author in [29].

The driving force quantities ${\left(\partial U_{ea}/\partial a\right)}_{X}$ and ${\left(\partial \Pi_{ea}/\partial a\right)}_{P}$ in (18) are equivalent to a parameter, ***I***, previously postulated by C.E. Turner and others as an elastic–plastic crack driving force [30,31,32]. To demonstrate this equivalence, graphical definitions of the partial derivatives ${\left(\partial U_{ea}/\partial a\right)}_{X}\;and\;{\left(\partial \Pi_{ea}/\partial a\right)}_{P}$ are shown in Figure 3 and Figure 4, respectively. Comparing these figures with the graphical definition of the parameter ***I*** given by the above authors [30,31,32] shows that quantities ***I***, ${\left(\partial U_{ea}/\partial a\right)}_{X}\;and\;{\left(\partial \Pi_{ea}/\partial a\right)}_{P}$ are identical.

Graphs such as those in Figure 3 and Figure 4 provide a means for the experimental determination of ***I*** from the measured areas under elastic unloading curves prior to and after an actual crack growth increment ∆a. This graphical method is quite general in that it allows for the measurement of ***I*** regardless of whether the elastic unloading curves are linear or non-linear, but it is subject to the usual errors involved in the numerical analysis of graphical data. In the typical case of linear elastic unloading behavior, a more convenient analytical method for calculating ***I*** is available in Formula (18) derived above, i.e.,

(19) $${I\equiv -\left(\frac{\partial U_{ea}}{\partial a}\right)}_{X}={\left(\frac{\partial \Pi_{ea}}{\partial a}\right)}_{P}=\left(\frac{1}{2}{\;P}^{2}\frac{dC}{da}+P\frac{dX_{o}}{da}\right)\;\;\;\;\;\;\;\;\;\;\;\;\;\;\;\;\;\;\;(0\leq P\leq P^{\prime })$$

All that is required for using (19) is plotting experimental values of C and $X_{o}$ against crack extension, calculating the derivatives $\frac{dC}{da}$ and $\frac{dX_{o}}{da}$ from the slopes of the corresponding curves (see Appendix A for an example of such calculations), and substituting the derivative values together with current load ***P*** into (19).

As emphasized by Turner [30], under elastic–plastic conditions the elastic–plastic crack driving force parameter ${I\equiv -\left(\frac{\partial U_{ea}}{\partial a}\right)}_{X}={\left(\frac{\partial \Pi_{ea}}{\partial a}\right)}_{P}$ differs fundamentally from the ***J*** integral. The parameter ***I*** fully admits crack extension accompanied by both plastic deformation and elastic unloading, whereas the ***J***-integral is undefined for such behavior. The two parameters become equivalent under fully elastic conditions. This can be noted for the general case of linear or non-linear elastic unloading, by inspecting the graphical definitions of ***I*** in Figure 3 and Figure 4. Under elastic conditions, all unloading curves intersect the origin, and residual elastic energy vanishes so that the apparent elastic energies $U_{ea}$ and $\Pi_{ea}$ become equivalent to the fully elastic parameters $U_{e.}$ and $\Pi_{e}$, respectively. This reduces Figure 3 and Figure 4 to the classical graphical definitions of the J-integral found in standard textbooks [9,10]. A similar result is obtained from the linearized analytical expression for ***I*** in (19). The plastic dissipation term $P\frac{dX_{o}}{da}$ vanishes under full elasticity, and the apparent elastic energies $U_{ea}$ and $\Pi_{ea}$ become equivalent to the fully elastic parameters $U_{e.}$ and $\Pi_{e}$, respectively. This reduces (19) to the well-known LEFM identity

(20) $${I\equiv -\left(\frac{\partial U_{e}}{\partial a}\right)}_{X}={\left(\frac{\partial \Pi_{e}}{\partial a}\right)}_{P}=\left(\frac{1}{2}{\;P}^{2}\frac{dC}{da}\right)=G$$

where ***G*** is Irwin’s crack driving force, equivalent to **J**.

#### 2.1.3. Energy Dissipation Rate D(a) and Associated Properties

The energy dissipation rate D(a), essentially a quasi-static relative of the Charpy and Izod impact parameters, has been used as a measure of crack growth resistance [31,32,33,34,35,36,37,38,39,40,41]. It is defined by the total derivative

(21) $$D(a)\equiv \left(\frac{d\left(W^{\prime }-U_{ea}^{\prime }\right)}{da}\right)=\left(\frac{P^{\prime }\left(a\right)dX^{\prime }\left(a\right)-dU_{ea}^{\prime }\left(a\right)}{da}\right)$$

where $dW^{\prime }\equiv \;P^{\prime }\left(a\right)dX^{\prime }\left(a\right)$ is the external work on a specimen required to extend the crack by an amount da, and $U_{ea}^{\prime }(a)$ is the apparent elastic energy released by unloading the body from the critical load $P^{\prime }(a)$ to the state P=0.

The above definition applies strictly for loading along the boldfaced tearing path in Figure 1 and Figure 2, where the specimen is always in a state of imminent tearing such that the load P and displacement X are always at their respective critical values $P^{\prime }(a)$ and $X^{\prime }\left(a\right)$. Therefore, all variables in (21) may be considered functions of crack length *a*, justifying the definition of ***D*** in the literature as a total derivative with respect to *a*. Note that this contrasts fundamentally with the parameter ***I*** defined by the partial derivative functions ${\left(\partial U_{ea}/\partial a\right)}_{X}\;and\;{\left(\partial \Pi_{ea}/\partial a\right)}_{P}$ in (19), which depends not only on current crack length *a* but also on the arbitrary loading state (X, P) on the corresponding elastic unloading curve.

A graphical definition of the total derivative $\frac{d\left(W^{\prime }-U_{ea}^{\prime }\right)}{da}$ is given in Figure 5, which can be used to determine ***D*** experimentally by measuring the indicated shaded area under the tearing curve. As in the case of driving force parameter ***I***, the graphical method for measuring ***D*** is general in that it admits both linear and non-linear elastic unloading curves. However, for linear elastic unloading behavior, a simpler analytical method can be used. Substituting the identities $U_{ea}^{\prime }\left(a\right)=1/2\;P^{\prime }\left(a\right)\cdot [X^{\prime }\left(a\right)-X_{o}\left(a\right)]$ and $P^{\prime }\left(a\right)=\left[X^{\prime }\left(a\right)-X_{o}\left(a\right)\right]/C(a)$ into (21), and performing the indicated differentiation, gives the following:

(22) $$D\left(a\right)=\frac{\frac{1}{2}{\left[X^{\prime }\left(a\right)-X_{o}\left(a\right)\right]}^{2}}{{C\left(a\right)}^{2}}\frac{dC}{da}+\frac{\left[X^{\prime }\left(a\right)-X_{o}\left(a\right)\right]}{C\left(a\right)}\frac{dX_{o}}{da}=\frac{1}{2}{P^{\prime }(a)}^{2}\frac{dC}{da}+P^{\prime }(a)\frac{dX_{o}}{da}$$

which is equivalent to a formula given by Turner and Kolednik [33] in their equation, Equation (3).

#### 2.1.4. Tearing Criterion Implicit in the Definitions of Parameters I and D

Inspection of (19) and (22) shows that, whenever the arbitrarily variable load P becomes equal to the critical load P’, ***and only then***, the following tearing criterion is satisfied:

(23) $$\;I\equiv {-\left(\frac{\partial U_{ea}}{\partial a}\right)}_{X}={\left(\frac{\partial \Pi_{ea}}{\partial a}\right)}_{P}=D$$

For convenience in subsequent discussion, (23) may be re-written by multiplying it by da as

(24) $$I\;da\equiv {-\left(\frac{\partial U_{ea}}{\partial a}\right)}_{X}da={\left(\frac{\partial \Pi_{ea}}{\partial a}\right)}_{P}da=D\;da$$

Criterion (23) was first derived analytically for specimens of unit thickness by the author in 2016 [29]. However, it should be noted that as far back as 1979, C.E. Turner ([30], p. 67) postulated the equivalent result (24) on heuristic grounds. For a fracture specimen of thickness B, he hypothesized that “*it is the total available rate B I da that must be balanced against the total dissipative work (B D da) for crack growth*” (the italics and expression in parentheses are the author’s). Unfortunately, Turner’s postulate never took hold. As shown above, his idea was not a mere postulate but rather a mathematical consequence of the observed structure of load–displacement diagrams.

### 2.2. Analysis of Elastic–Plastic Materials with Non-Linear Elastic Unloading Behavior

In the above section, it was shown that tearing criteria (23) and (24) can be derived analytically for elastic–plastic specimens with linear elastic unloading behavior. Such an analysis is impossible for non-linear elastic unloading curves due to the infinite number of shapes that such curves can assume. However, tearing criteria (23) and (24) can still be demonstrated with reasonable rigor for non-linear elastic unloading, using graphical analysis.

We first refer back to Figure 3 and Figure 4, comparing graphical definitions of the fixed-grips and fixed-load driving force derivatives. In the caption of Figure 4, it was pointed out that the graphical difference between fixed-grips and fixed-load parameters ${\left(\frac{\partial U_{ea}}{\partial a}\right)}_{X}$ and ${\left(\frac{\partial \Pi_{ea}}{\partial a}\right)}_{P}$ was restricted to the small triangular area *bfe* in Figure 4. This area vanishes in the limit ∆a→0, rendering the derivatives ${\left(\frac{\partial U_{ea}}{\partial a}\right)}_{X}$ and ${\left(\frac{\partial \Pi_{ea}}{\partial a}\right)}_{P}$ equivalent. The same result applies regardless of whether the elastic unloading curves in Figure 3 and Figure 4 are linear or non-linear.

Having established the equivalence of driving forces $-{\left(\frac{\partial U_{ea}}{\partial a}\right)}_{X}$ and ${\left(\frac{\partial \Pi_{ea}}{\partial a}\right)}_{P}$ for elastic-plastic specimens with non-linear elastic unloading behavior, we next use the graphical inspection method to demonstrate equality of these parameters to the energy dissipation rate D at imminent crack extension. To do this, we compare the graphical illustration of D in Figure 5 to a graphical illustration of the fixed-load driving force ${\left(\frac{\partial \Pi_{ea}}{\partial a}\right)}_{P}$ at imminent fracture (Figure 6). Note that Figure 6 differs from the graphical definition of D in Figure 5 only by the small area painted in gray in Figure 6.

As explained in the caption of Figure 6, this small area vanishes in the limit ∆a→0, rendering ${\left(\frac{\partial \Pi_{ea}}{\partial a}\right)}_{P}$ and **D** equivalent. Identity with the fixed grips driving force then follows from the previously established equivalence $-{\left(\frac{\partial U_{ea}}{\partial a}\right)}_{X}={\left(\frac{\partial \Pi_{ea}}{\partial a}\right)}_{P}$. In conclusion, then, we can state that *tearing criteria (23) and (24) apply equally well to specimens with either linear or non-linear elastic unloading behavior*.

## 3. Thermodynamic Tearing Criterion

The above energy criteria (23) and (24) are essentially empirical results, arising from inspection of experimental load–displacement diagrams. In their derivation, no reference at all was made to thermodynamic concepts. In what follows, the problem is approached from the opposite direction, parting from a purely thermodynamic perspective where nothing is assumed a priori other than existence of a thermodynamic state function of form U(∄, X, S, *a*), subject to the first and second laws of thermodynamics. The thermodynamic formulation will be shown to yield a generalized elastic-plastic fracture criterion admitting both linear and non-linear elastic unloading behavior, *which turns out to be identical* to empirical criterion (24). This provides the desired empirical verification of the thermodynamic criterion.

### 3.1. Conventions in the Thermodynamic Model

The convention is adopted that any heat added to a body is a positive quantity, whereas heat transferred out of the body is negative. Similarly, work performed by the environment on the body, i.e., “loading work”, is positive, whereas working by the body on the environment or “unloading work” is negative. A load-point displacement dX is always assigned the same algebraic sign as that of the work increment dW≡PdX which it produces, so that dX is always positive for loading processes and negative for unloading. Under this convention P is always positive. For simplicity, a cracked body is considered as a closed system, subject to work and heat exchange with the environment but no mass exchange (no chemical reactions occur between exposed crack surfaces and environmental substances). Time independent conditions are assumed throughout, allowing Gibbs’ [19] convention of expressing parameter variations in time-implicit form.

### 3.2. Thermodynamic State Function and Associated Derivative Relations

The parting point for analysis is the assumed state function

(25) U=U(∄,S, X, a)

where all variables have been defined previously. For a fracture test specimen of given loading configuration (fixed ∄), expansion of (25) in terms of partial derivatives gives

(26) $$dU={\left(\frac{\partial U}{\partial X}\right)}_{a,S}dX+{\left(\frac{\partial U}{\partial S}\right)}_{X,a}dS+{\left(\frac{\partial U}{\partial a}\right)}_{X,S}da$$

An important restriction on (26) must be noted, pertaining to fracture specimens with pre-cut notches of the type commonly used to measure the elastic-plastic J-integral. During the initial plastic crack tip blunting stage prior to fracture initiation, the crack tip remains stationary so that internal irreversible processes cannot be related to changing crack length, rendering (26) invalid. In that case, a better macroscopic measure of internal irreversible processes might be an observable integral of the plastic deformation field, such as crack tip opening displacement (CTOD). It is only once post-yield tearing begins, with characteristic formation of a sharp propagating crack tip [10], that constitutive linkage between internal irreversible processes and crack extension can be assumed, admitting (26). Therefore, the analysis here is restricted to growing cracks.

### 3.3. Constraints Imposed by the First and Second Laws of Thermodynamics

The first law of thermodynamics imposes that the internal energy change dU of a specimen undergoing a thermomechanical process is subject to

(27) $$dU=dW+dq=PdX+TdS-dq_{dis}$$

The differential dW is the work performed by external forces, dq is the heat exchanged between the specimen and the environment, dS is the entropy change in the specimen and $dq_{dis}$ is the dissipation defined in Truesdell [42] as:

(28) $$dq_{dis}\equiv TdS-dq\geq 0$$

where the inequality is imposed by the second law of thermodynamics.

Consistency of (26) with the first and second law constraints (27) and (28) requires that ${\left(\frac{\partial U}{\partial X}\right)}_{a,S}=P,\;{\;\left(\frac{\partial U}{\partial S}\right)}_{X,a}=T$ and $-{\left(\frac{\partial U}{\partial a}\right)}_{X,S}da=dq_{dis}\geq 0$. Making use of the first two of these identities, state function (26) can be written as

(29) $$dU=PdX+TdS+{\left(\frac{\partial U}{\partial a}\right)}_{X,S}da$$

For isothermal conditions, simplification is achieved by substituting the identity $d\left(TS\right)=TdS+SdT$ together with the isothermal condition dT=0 into (29), yielding

(30) $$dA=PdX+{\left(\frac{\partial U}{\partial a}\right)}_{X}da=\;{\left(\frac{\partial A}{\partial X}\right)}_{a}dX+{\left(\frac{\partial A}{\partial a}\right)}_{X}da=dW+{\left(\frac{\partial A}{\partial a}\right)}_{X}da$$

where A≡U−TS is the Helmholtz energy and the identities ${\left(\frac{\partial A}{\partial X}\right)}_{a}=P$ and ${\left(\frac{\partial U}{\partial a}\right)}_{X}da={\left(\frac{\partial A}{\partial a}\right)}_{X}da$ are implicit. Making use of the second of these identities and introducing the earlier result ${-\left(\frac{\partial U}{\partial a}\right)}_{X,S}da=dq_{dis}\geq 0$ into (30) gives, after rearrangement,

(31) $$-{\left(\frac{\partial A}{\partial a}\right)}_{X}da=dW-dA=dq_{dis}\geq 0$$

This constitutes a thermodynamic condition on both reversible and irreversible processes in fracture specimens. For reversible unloading and re-loading at fixed crack length, both d*a* and $dq_{dis}$ vanish, reducing (31) to the result dA=dW. On the other hand, for irreversible tearing processes where d*a* and $dq_{dis}$ are generally non-vanishing, and where load coordinates (X, P) are always at the critical values (X’, P’), (31) becomes

(32) $$-{\left(\frac{\partial A}{\partial a}\right)}_{X}da=dW^{\prime }-dA^{\prime }=dq_{dis}\geq 0$$

where again the prime notation denotes processes restricted to the tearing path.

The domain of (32) can be extended beyond the fixed-grips condition to also admit fixed load P, by substituting the identity $d\left(PX\right)=PdX+XdP$ into (30) yielding

(33) $$d\left(PX-A\right)=d\Pi \left(P,a\right)=-{\left(\frac{\partial A}{\partial a}\right)}_{X}da+XdP={\left(\frac{\partial \Pi }{\partial a}\right)}_{P}da+{\left(\frac{\partial \Pi }{\partial P}\right)}_{a}dP$$

where $\Pi \left(P,a\right)\equiv PX-A$ is the complementary energy. Comparing coefficients of the d*a* in (33) gives the identity

(34) $$-{\left(\frac{\partial A}{\partial a}\right)}_{X}={\left(\frac{\partial \Pi }{\partial a}\right)}_{P}$$

which substituted into (32) gives the thermodynamic condition for tearing under either fixed grips or fixed load as

(35) $$-{\left(\frac{\partial A}{\partial a}\right)}_{X}da={\left(\frac{\partial \Pi }{\partial a}\right)}_{P}da=dW^{\prime }-dA^{\prime }=dq_{dis}\geq 0$$

Criterion (35) is essentially Rice’s criterion (4) from the Introduction, generalized to admit fixed-load conditions and incorporating the additional quantity $dW^{\prime }-dA^{\prime }$.

### 3.4. Empirical Verification of Thermodynamic Constraint

In the following, experimental verification of thermodynamic criterion (35) is achieved by showing its equivalence to empirical energy criterion (24).

The first step is relating the energy parameters A and Π in (35) to the parameters $U_{ea}$ and $\Pi_{ea}$ in (24), making use of the Helmholtz energy expression (30), which indicated that

(36) $${\left(\frac{\partial A}{\partial X}\right)}_{a}=P$$

This shows that the derivative ${\left(\partial A/\partial X\right)}_{a}$ vanishes in the unloaded state P=0, admitting a non-vanishing value of A at P=0 that is at most some indeterminate function of crack length *a*. This function is designated as the “residual Helmholtz energy”$A_{o}(a)$. Similar indeterminacy of A in elastic–plastic materials was pointed out by Hill and Rice [43], who observed that under isothermal conditions, A could at most be specified to within some unknown function **H** of the set of internal variables. Formally, the residual Helmholtz energy component $A_{o}$ is simply a mathematical consequence of constraint (36). However, $A_{o}$ can also be given a physical interpretation as the set containing all Helmholtz energy forms that are not recovered as work on the environment when external loads are removed. This admits non-dissipative irreversible energy forms of the type described earlier, such as residual elastic energy and Griffith surface energy. It does not include thermal dissipation energy, which has already been accounted for in the parameter $q_{dis}$.

Integrating (36) with respect to X at fixed *a*, parting from the plastic set point ${X=X}_{o}\left(a\right)$ where P=0 and $A=A_{o}\left(a\right)$, gives A(X,a) as

(37) $$A\left(X,a\right)=A_{o}\left(a\right)+\int_{X_{o}\left(a\right)}^{X}P\left(\tau ,a\right)d\tau =A_{o}\left(a\right)+U_{ea}\left(X,a\right)$$

where the integral $\int_{X_{o}\left(a\right)}^{X}P\left(\tau ,a\right)d\tau$ defines the apparent elastic energy $U_{ea}\left(X,a\right)$. It should be noted that the integral definition of $U_{ea}\left(X,a\right)$ in (37) ***admits both linear and non-linear elastic unloading***, containing the linear compliance-based definition of $U_{ea}\left(X,a\right)$ in (10) as a special case. Identity (37) provides a relation between the Helmholtz energy A and the residual elastic energy $U_{ea}$. It represents a generalization of Rice’s LEFM constraint (5) on the Helmholtz energy mentioned in the Introduction. Rice’s constraint constituted a special case of (37) where $A_{o}\left(a\right)=2\gamma a$ and $U_{ea}\left(X,a\right)=U_{e}\left(X,a\right)$.

Thermodynamic constraint (35) can now be transformed into an equivalent form in terms of the elastic–plastic parameters $U_{ea}\left(X,a\right)$ and $\Pi_{ea}(P,a)$. Substituting identity (37) into the individual parameters ${\left(\frac{\partial A}{\partial a}\right)}_{X}$, ${\left(\frac{\partial \Pi }{\partial a}\right)}_{P}$ and dA’ of thermodynamic constraint (35) gives the parameters

(38) $${\left(\frac{\partial A}{\partial a}\right)}_{X}={\left(\frac{\partial U_{ea}}{\partial a}\right)}_{X}+\frac{dA_{o}}{da}$$

(39) $${\left(\frac{\partial \Pi }{\partial a}\right)}_{P}={\left(\frac{\partial (PX-A)}{\partial a}\right)}_{P}={\left(\frac{\partial (PX-{[U}_{ea}+A_{o})}{\partial a}\right)}_{P}={\left(\frac{\partial \Pi_{ea}}{\partial a}\right)}_{P}-\frac{dA_{o}}{da}$$

and

(40) $${\mathrm{dA}}^{\prime }={{dU}_{ea}}^{\prime }+d\;A_{o}$$

Introducing identities (38)–(40) back into thermodynamic criterion (35) yields the *equivalent thermodynamic criterion*

(41) $$-{\left(\frac{\partial U_{ea}}{\partial a}\right)}_{X}da={\left(\frac{\partial \Pi_{ea}}{\partial a}\right)}_{P}da=\left(dW^{\prime }-d{Uea}^{\prime }\right)=\;dq_{dis}+dA_{o})$$

or, making use of the definition of **D** in (21),

(42) $$-{\left(\frac{\partial U_{ea}}{\partial a}\right)}_{X}da={\left(\frac{\partial \Pi_{ea}}{\partial a}\right)}_{P}da=Dda=dq_{dis}+dA_{o}$$

It is immediately evident that thermodynamic criterion (42) includes the identity $-{\left(\frac{\partial U_{ea}}{\partial a}\right)}_{X}da={\left(\frac{\partial \Pi_{ea}}{\partial a}\right)}_{P}da=D\;da$, which is simply the empirical energy criterion (24). This provides the desired empirical evidence for the validity of thermodynamic criterion (42), and, conversely, shows that the empirically obtained relation (24) is actually a thermodynamic criterion.

However, an additional piece of information arises in thermodynamic criterion (42), which does not appear in the conventional definitions of **D** in (21) and (22) or in the empirical energy criterion (24). This is the identity $D\;da=dq_{diss}+dA_{o}$, giving D a rigorous thermodynamic definition as $D\equiv \frac{\left(dq_{diss}+dA_{o}\right)}{da}$, equating it to the sum of heat dissipated per unit crack extension plus the rate of change in the residual Helmholtz energy $A_{o}$. In the past, D was treated largely as an empirical parameter of uncertain theoretical significance, which probably explains why it is rarely, if ever, mentioned in fracture mechanics textbooks. The result obtained above clarifies the issue by giving D a rigorous thermodynamic interpretation.

The identity $D\equiv \frac{\left(dq_{diss}+dA_{o}\right)}{da}$ also has potential experimental implications. Note that the thermodynamic definition $D\equiv \frac{\left(dq_{diss}+dA_{o}\right)}{da}$, together with the conventional definitions of D in (21) or (22), constitute a set of two independent equations from which the variable D can be eliminated. Assuming sufficiently accurate calorimetric methods for measuring the thermal dissipation $dq_{dis}$, these two equations can then in principle be solved for the change ${dA}_{o}$ in residual Helmholtz energy associated with a crack growth increment d*a*.

It should be noted that the inequality condition ≥0 in thermodynamic criterion (35) has been removed from the equivalent thermodynamic relation (42). This is in response to the fact that **D** in (42) represents the sum of dissipated heat $dq_{dis}$ plus the change ${dA}_{o}$ in residual Helmholtz energy. The term $dq_{dis}$ is always positive by virtue of the second law of thermodynamics, but no such general law has been established for ${dA}_{o}$. This allows D to be negative in principle, although experimental studies [29,30,31,32,33,34,35,36,37,38,39] have found it to be generally positive.

## 4. Applications of the Thermodynamic Criterion

### 4.1. Fully Elastic Fracture

In the conceptual model of fully elastic fracture as a non-dissipative process, the dissipation term $dq_{diss}$ vanishes and the driving force partial derivatives $-{\left(\frac{\partial U_{ea}}{\partial a}\right)}_{X}$ and ${\left(\frac{\partial \Pi_{ea}}{\partial a}\right)}_{P}$ reduce to the elastic parameters $-{\left(\frac{\partial U_{e}}{\partial a}\right)}_{X}$ and ${\left(\frac{\partial \Pi_{e}}{\partial a}\right)}_{P}$, which are equivalent to the elastic J-integral. This reduces thermodynamic criterion (42) to the condition $J=D=\frac{dA_{o}}{da}$.

### 4.2. Griffith and Irwin Fracture Criteria as Special Cases of the Thermodynamic Criterion

As noted above, the residual Helmholtz energy term admits non-dissipative irreversible energy forms such as residual elastic energy ($U_{er})$ and Griffith surface energy (2γa). We may correspondingly write

(43) $$A_{o}\left(a\right)=U_{er}\left(a\right)+2\gamma a+\xi (a)$$

where ξ(a) is a miscellaneous term accounting for any forms of $A_{o}\left(a\right)$ other than $U_{er}\left(a\right)$ and 2γa. Introducing (43) into the right-hand side of thermodynamic criterion (42), and letting the miscellaneous energy term ξ(a) vanish, (42) becomes

(44) $$-{\left(\frac{\partial U_{ea}}{\partial a}\right)}_{X}da={\left(\frac{\partial \Pi_{ea}}{\partial a}\right)}_{P}da=Dda=dq_{diss}+dA_{o}=dq_{diss}+2\gamma da+dU_{er}\left(a\right)$$

We next write the total elastic energy $U_{e}$ as the sum of the apparent elastic energy $U_{ea}\left(a,X\right)$ and residual elastic energy $U_{er}\left(a\right)$:

(45) $$U_{e}\left(a,X\right)\equiv U_{ea}\left(a,X\right)+U_{er}\left(a\right)$$

Substituting [44] into [45] yields

(46) $$-\left[{\left(\frac{\partial U_{e}}{\partial a}\right)}_{X}-\frac{dU_{er}}{da}\right]da=\left[{\left(\frac{\partial \Pi_{e}}{\partial a}\right)}_{P}+\frac{dU_{er}}{da}\right]da=dq_{diss}+2\gamma da+dU_{er}\left(a\right)\approx dW_{p}+2\gamma da+dU_{er}\left(a\right)$$

All terms in $dU_{er}$ cancel out from (46), reducing it to

(47) $$-{\left(\frac{\partial U_{e}}{\partial a}\right)}_{X}da={\left(\frac{\partial \Pi_{e}}{\partial a}\right)}_{P}da=dW_{p}+2\gamma da$$

which is Irwin’s postulate (2). Griffith’s criterion (1) is simply the special case of (47) where the plastic work $dW_{p}$ vanishes.

### 4.3. Relation of Thermodynamic Criterion to Crack Tip Stress Intensity Ahead of Growing Cracks

In the common case of elastic–plastic fracture specimens with linear elastic unloading behavior, thermodynamic fracture criterion (42) may be written in linearized form as

(48) $$I={-\left(\frac{\partial U_{ea}}{\partial a}\right)}_{X}={\left(\frac{\partial \Pi_{ea}}{\partial a}\right)}_{P}=D=\frac{1}{2}{P^{\prime }}^{2}\frac{dC}{da}+P^{\prime }\frac{dX_{o}}{da}$$

This yields some insight into relations between energy derivatives and crack tip stress intensity during quasi-static crack extension. Assuming small-strain linear elastic unloading behavior of a specimen subjected to external load P, a linear elastic stress–strain field can be defined, with the corresponding material reference configuration being the final unloaded state (P=0) of the specimen. This field represents an excess elastic field relative to any residual stress-deformation field remaining in the specimen in its final unloaded state [6]. The excess field is identical to the K-dominated stress–strain field in an LEFM specimen with the same loading configuration, crack length and elastic modus E’ as the elastic-plastic specimen. This allows applying the LEFM identity ${K^{\prime }}^{2}/E^{\prime }=\frac{1}{2}{\;P^{\prime }}^{2}dC/da$ to elastic-plastic specimens, forming the basis of well-known unloading compliance methods for determining crack length and the so-called “elastic” component ${K^{\prime }}^{2}/E\prime$ of the J-integral in elastic-plastic specimens [9,10]. The same identity ${K^{\prime }}^{2}/E\prime =\frac{1}{2}{\;P^{\prime }}^{2}dC/da$ may be substituted into the linearized elastic-plastic tearing criterion (48), giving

(49) $$I={-\left(\frac{\partial U_{ea}}{\partial a}\right)}_{X}={\left(\frac{\partial \Pi_{ea}}{\partial a}\right)}_{P}=D=\frac{{K^{\prime }}^{2}}{E^{\prime }}+P^{\prime }\frac{dX_{o}}{da}$$

This provides a relationship between the thermodynamic driving force ***I*** (or resistance ***D***) and an evolving K-dominated excess elastic field ahead of growing cracks.

The significance of (49) may perhaps be questioned on grounds that K only takes into account the excess elastic field rather than the total stress field ahead of a crack tip. However, two counter-arguments are offered. First is the fact that the K-dominated field arises naturally in the thermodynamic formulation and is a characteristic crack tip parameter, relatable to the applied load P for various system configurations by means of available LEFM solutions. Secondly, even though K only represents an excess field, this does not necessarily imply that the excess field is small relative to the total stress field. In some cases the two fields may be nearly identical. For example, Snyder and Miller [45] found that the measured tensile strength of highly plastic moist soil could be predicted by assuming theoretical LEFM (total) tensile stress distributions around crack tips, superimposed on a uniform cohesive stress field caused by capillary pore-water suction. This result would be difficult to explain if significant indeterminate residual stress fields were influencing tensile strength. Similarly, measured stress distributions in soil beneath loaded surfaces have been found to agree reasonably well with linear elastic solutions based on the Boussinesq equation [44].

A prediction of (49) is that in elastic–plastic materials, the driving force derivatives (or the resistance D) are non-uniquely related to the excess crack tip stress field, due to presence of the independent dissipative term $P^{\prime }\frac{dX_{o}}{da}$. Uniqueness is only predicted in the limit of LEFM behavior, where the residual elastic energy and the dissipation term vanish, reducing (49) to the LEFM relation

(50) $$I={-\left(\frac{\partial U_{e}}{\partial a}\right)}_{X}={\left(\frac{\partial \Pi_{e}}{\partial a}\right)}_{P}=D=\frac{1}{2}{P^{\prime }}^{2}\frac{dC}{da}=\frac{{K^{\prime }}^{2}}{E^{\prime }}$$

The non-unique relation (49) between stress intensity and driving or resistance forces in elastic–plastic specimens illustrates the difficulties in attempting to characterize the tearing of elastic–plastic materials by means of a single parameter. A dual set of parameters, characterizing both stress intensity and energy requirements per unit crack extension (i.e., toughness), would seem more appropriate. Possible sets could include K (or J for incipient crack growth) as a stress intensity parameter, and D as a measure of toughness.

### 4.4. Stability Considerations

Orowan (4) established the principle that fracture instability ensues whenever the rate of change in driving force per unit crack extension equals or exceeds the rate of change in resistance force. Applying this principle to thermodynamic tearing criterion (42) under fixed-grips gives the instability condition, which is as follows:

(51) $$-{\left(\frac{\partial^{2}U_{ea}}{\partial a^{2}}\right)}_{X}\geq \frac{dD}{da}\;\;\;\;\;\;\;\;\;\;\;\;\;\;\;(fixed\;grips)$$

The corresponding condition for fixed-load is

(52) $${\left(\frac{\partial^{2}\Pi_{ea}}{\partial a^{2}}\right)}_{P}\geq \;\frac{dD}{da}\;\;\;\;\;\;\;\;\;\;\;\;\;\;\;\;\;\;(fixed\;load)$$

where in general, $-{\left(\frac{\partial^{2}U_{ea}}{\partial a^{2}}\right)}_{X}\neq {\left(\frac{\partial^{2}\Pi_{ea}}{\partial a^{2}}\right)}_{P}$.

Application of these criteria requires knowledge of the functional dependence of the various energy parameters on crack length and load coordinates, which, in general, entails detailed experimental measurements. However, considerable simplification is possible in the special common case of materials with linear elastic unloading behavior. For this case, substituting linearized expressions (13) and (22) for ${\left(\frac{\partial U_{ea}}{\partial a}\right)}_{X}$ and D into fixed grips inequality (51), and performing the indicated differentiation, gives the fixed grips instability condition:

(53) $$\;\frac{dP^{\prime }}{da}\leq -\frac{1}{C}\left(P^{\prime }\frac{dC}{da}+\frac{dX_{o}}{da}\right)\;\;\;\;\;\;\;\;\;\;\;\;\;\;\;\;\;\;\left(fixed\;grips\right)$$

Please note that the parameters C, P’, $\frac{dC}{da}$ and $\frac{dX_{o}}{da}$ are either vanishing or positive, so that the quantity $-\frac{1}{C}\left(P^{\prime }\frac{dC}{da}+\frac{dX_{o}}{da}\right)$ on the right hand side of (53) can only be ≤0.

For the fixed-load instability condition, substituting linearized expressions (17) and (22) for ${\left(\frac{\partial \Pi_{ea}}{\partial a}\right)}_{P}$ and D into inequality (51) yields the instability criterion

(54) $$\left(P^{\prime }\frac{dC}{da}+\frac{dX_{o}}{da}\right)\frac{dP^{\prime }}{da}\leq 0\;\;\;\;\;\;\;\;\;\;\;\;\;\;\;\;\;\;\;\;\left(fixed\;load\right)$$

or, noting that the factor $\left(P^{\prime }\frac{dC}{da}+\frac{dX_{o}}{da}\right)$ is, in general, positive,

(55) $$\frac{dP^{\prime }}{da}\leq 0\;\;\;\;\;\;\;\;\;\;\;\;\;\;\;\;\;\;\;\;\left(fixed\;load\right)$$

Instability conditions (53) and (55) allow the following predictions on the stability of fracture toughening and fracture weakening systems under load-controlled or displacement-controlled conditions to be made.

*Fracture toughening systems*, characterized by positive slope $dP^{\prime }/da$ of the tearing path, always violate both of the fixed grips and fixed load instability criteria (53) and (55). These systems are therefore predicted to be stable.

*Fracture-weakening systems*, characterized by negative slope $dP^{\prime }/da$ of the tearing path, are predicted to be either stable or unstable, depending on the magnitude of $dP^{\prime }/da$ and whether displacement-controlled (fixed grips) or load-controlled (fixed load) conditions prevail. Under load-controlled conditions, these systems always satisfy criterion (55) and are therefore predicted to be unstable. But under displacement-controlled conditions, the corresponding criterion (53) predicts that instability occurs ***only if*** $dP^{\prime }/da$ is more negative than the critical fixed-grips instability parameter $-\frac{1}{C}\left(P^{\prime }\frac{dC}{da}+\frac{dX_{o}}{da}\right)$, which in itself is a negative number. This means that under displacement control, fracture weakening systems are stable so long as the loading slope $dP^{\prime }/da$ is less negative than $-\frac{1}{C}\left(P^{\prime }\frac{dC}{da}+\frac{dX_{o}}{da}\right)$, but become unstable for more negative loading slopes. The prediction is qualitatively consistent with the empirical observation [9] that fracture-weakening systems under displacement control can be stable within a certain loading range, beyond which instability sets in.

The above distinctions between the stability of fracture-toughening and fracture-weakening systems under displacement and load control are summarized in Figure 7. Similar behavior has been indicated elsewhere (see, for example Figure 9.14 in Anderson [9]). However, a distinguishing property of Figure 7 is the specific quantitative condition $dP^{\prime }/da\leq -\frac{1}{C}\left(P^{\prime }\frac{dC}{da}+\frac{dX_{o}}{da}\right)$ marking the onset of fixed-grips instability. Establishing whether or not this condition applies generally will require detailed experimentation and/or a review of existing data, including specimens with different loading configurations, involving materials with variable degrees of fracture toughening/softening. Such a research program is beyond the scope of the present paper, which is limited to outlining basic concepts. However, to illustrate the experimental methodology used for evaluating the stability criterion, a preliminary test using published data is presented in the Appendix A.

## 5. Limitations of the Theory

As emphasized by the qualifying term “quasi-static” in the title, an important assumption of the theory presented here is time independence. As such, it does not apply in the presence of time-dependent phenomena such as creep, fatigue-induced fracture, or inertial/viscous reaction to high loading rates. Another assumption, implicit in conservation constraint (36) of the thermodynamic formulation, is that no dissipation occurs during an unloading and reloading cycle at a fixed crack length. What this means in practical terms is that the extent of dissipation during an unloading or re-loading process must at most be small relative to the change in reversible (elastic) energy during that process. In a load–displacement diagram, this translates into the requirement that the area inside a hysteresis loop formed during unloading and re-loading (area *abcda* in Figure 8) must be small relative to the area under the “average” unloading and re-loading curve (area *ace* in Figure 8). Many standard methods for fracture toughness [27] assume this type of behavior.

## 6. Summary and Conclusions

A macroscopic thermodynamic criterion has been formulated for the tearing of elastic and elastic–plastic fracture specimens under isothermal quasi-static conditions. Underlying assumptions are the first and second laws of thermodynamics, and an internal energy function where the independent variables are load-point displacement, entropy and crack extension, with the latter assuming the role of internal state variable. The criterion takes the form ***I*** = ***D***, where ***I*** is a generalized driving force that reduces to the J-integral in the fully elastic domain and ***D*** is the energy dissipation rate, identified thermodynamically as the sum of heat dissipated per unit crack extension plus the rate of change in a residual Helmholtz energy parameter admitting non-dissipative irreversible energy forms such as residual elastic energy and Griffith surface energy. The criterion is applicable across the entire material behavioral spectrum ranging from fully elastic to highly ductile elastic–plastic, and, unlike LEFM or J-integral theory, fully accommodates extended crack growth accompanied by simultaneous plastic deformation and elastic unloading. Verification of the themodynamic criterion is obtained by showing its equivalence to energy relations implicit in the empirical structure of load displacement diagrams.

The thermodynamic formulation gives parameter D a rigorous thermodynamic definition as the sum of heat dissipated per unit crack extension plus the rate of change of a residual Helmholtz energy parameter admitting non-dissipative irreversible energy forms such as residual elastic energy and Griffith surface energy. It also suggests an experimental methodology for differentiating between the relative amounts of dissipative and non-dissipative irreversible energy forms generated in fracture.

In the special yet common case of elastic plastic materials exhibiting linear elastic unloading behavior, the respective crack driving and resistance forces I and D are representable as the sum of a dissipative term and the term $\frac{{K^{\prime }}^{2}}{E^{\prime }}$ describing an excess elastic stress field preceding growing cracks. This negates the possibility of a unique relation between thermodynamic forces I or D and the stress field surrounding a growing crack, except in the LEFM case where the dissipation term vanishes. An important experimental implication is that elastic–plastic fracture characterization requires, as a minimum, two parameters, one accounting for stress intensity and another accounting for toughness or energy expenditure per unit crack extension.

Fixed-grips and fixed-load instability criteria are postulated, which in the linearized case reduce to simple inequalities between system parameters. The criteria predict qualitative differences that have been empirically observed for stability under displacement- vs. load-controlled conditions. Application to a published experimental data set (see Appendix A) showed that predicted stability conditions were always satisfied during stable crack growth. The predicted condition for instability was very nearly approached at the point where further loading and crack extension were terminated.

Finally, it is shown that the original energy criteria for fracture postulated by Griffith and Irwin are essentially equivalent to the thermodynamic formulation under a variable transformation.

Important assumptions of the theory are time-independent conditions, and negligible dissipation during unloading and re-loading cycles at fixed crack length.

## Figures and Tables

**Figure 1:**
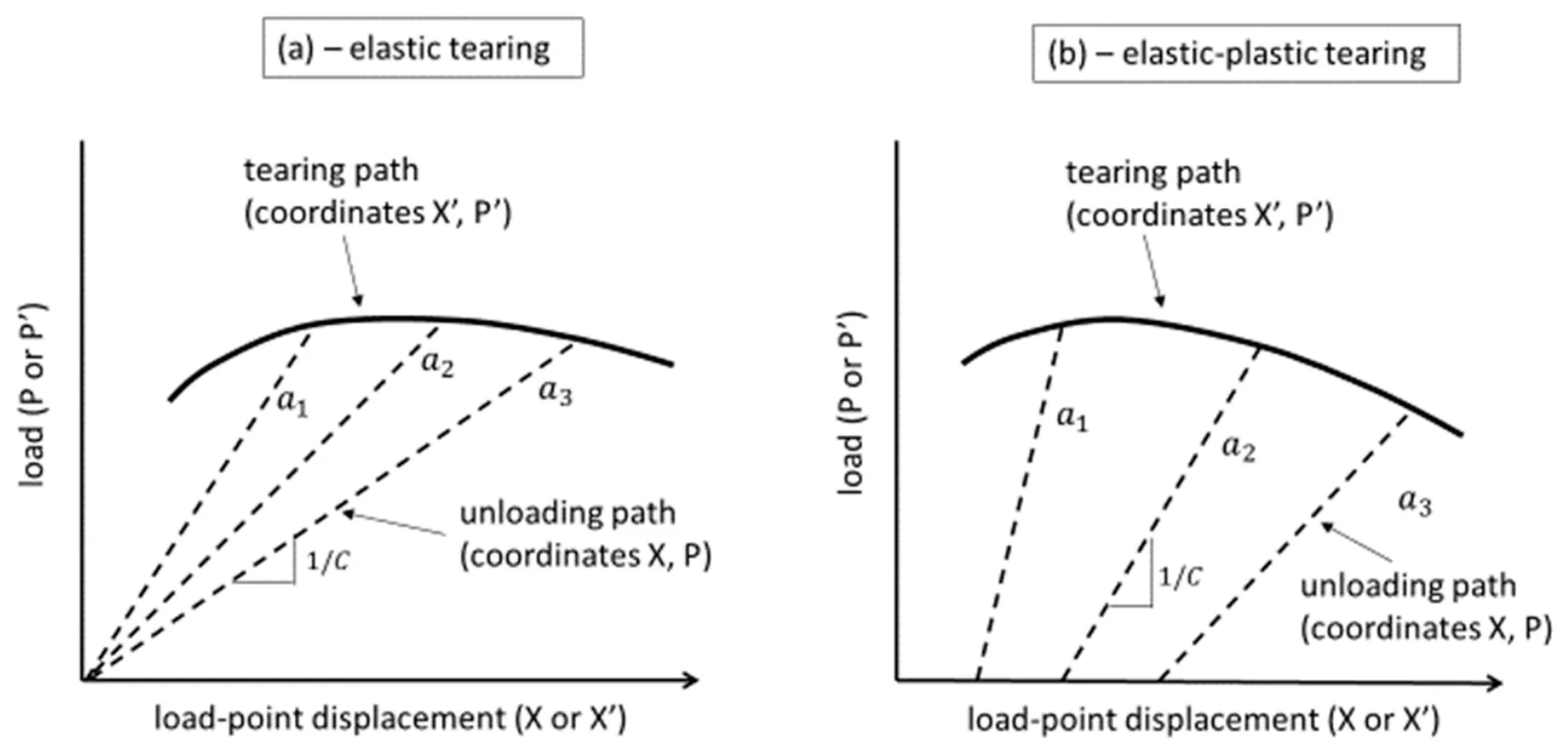
Structure of load–displacement diagrams obtained for elastic and elastic–plastic fracture specimens.

**Figure 2:**
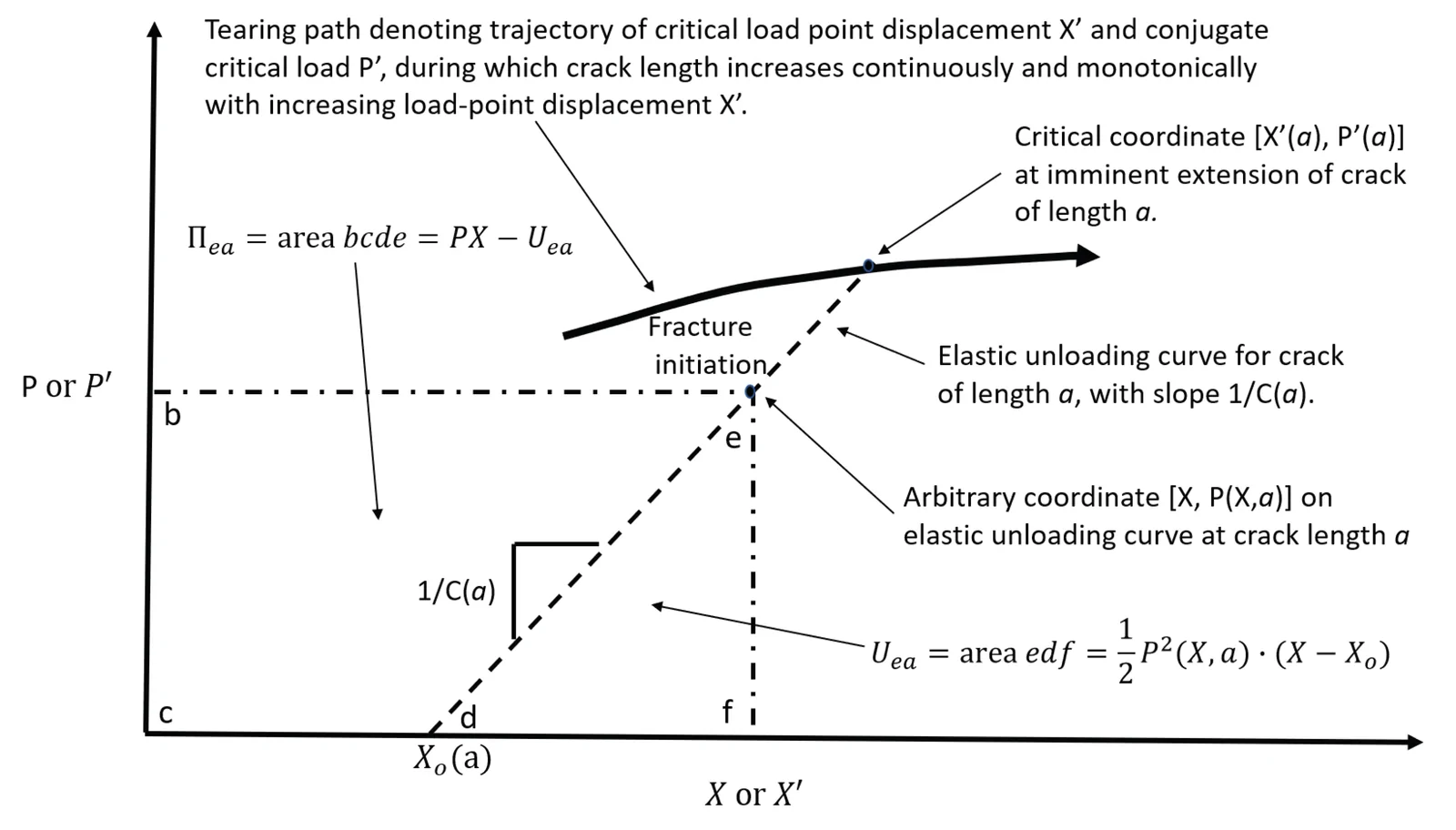
Load–displacement diagram illustrating (1) tearing path (bold-faced curve); (2) elastic unloading path corresponding to crack length a (dashed line); (3) unloading compliance *C*(*a*); (4) plastic set $X_{o}\left(a\right)$; (5) apparent elastic unloading energy $U_{ea}(X.a)$ and apparent complementary energy $\Pi_{ea}\left(P,a\right)$.

**Figure 3:**
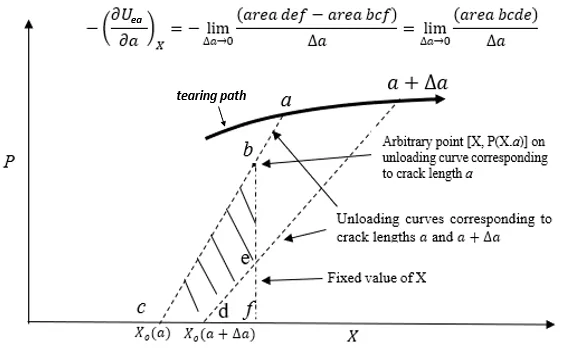
Graphical definition of fixed grips derivative ${\left(\partial U_{ea}/\partial a\right)}_{X}$ for arbitrary point b on elastic unloading curve corresponding to crack length a.

**Figure 4:**
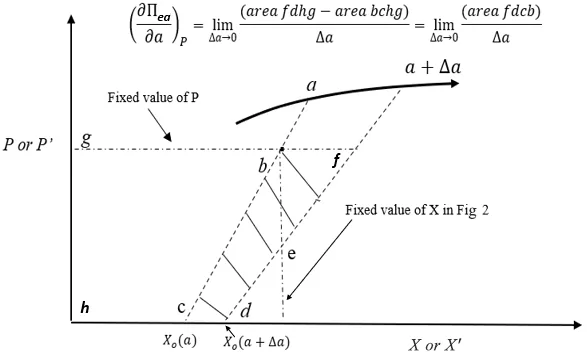
Graphical definition of fixed load partial derivative ${\left(\partial \Pi_{ea}/\partial a\right)}_{P}$ for the same arbitrary loading point b as in Figure 3. The dotted horizontal line is the fixed value of P, and the dotted vertical line is the same fixed-X line of Figure 2. Note that the shaded area in Figure 4 exceeds that of Figure 3 only by the triangular area bfe of Figure 4. This area, exhibiting second-order variation in ∆P∆X, vanishes faster with decreasing ∆a than area bcde, which exhibits first-order variation in $\left\|\vec{cb}\right\|\cdot ∆X.$ In the limit ∆a→0, area bfe in Figure 4 effectively vanishes, rendering the shaded areas in Figure 3 and Figure 4 equivalent as required by the analytical result (18). Please note that the above graphical analysis applies even if the elastic unloading curves happen to be non-linear.

**Figure 5:**
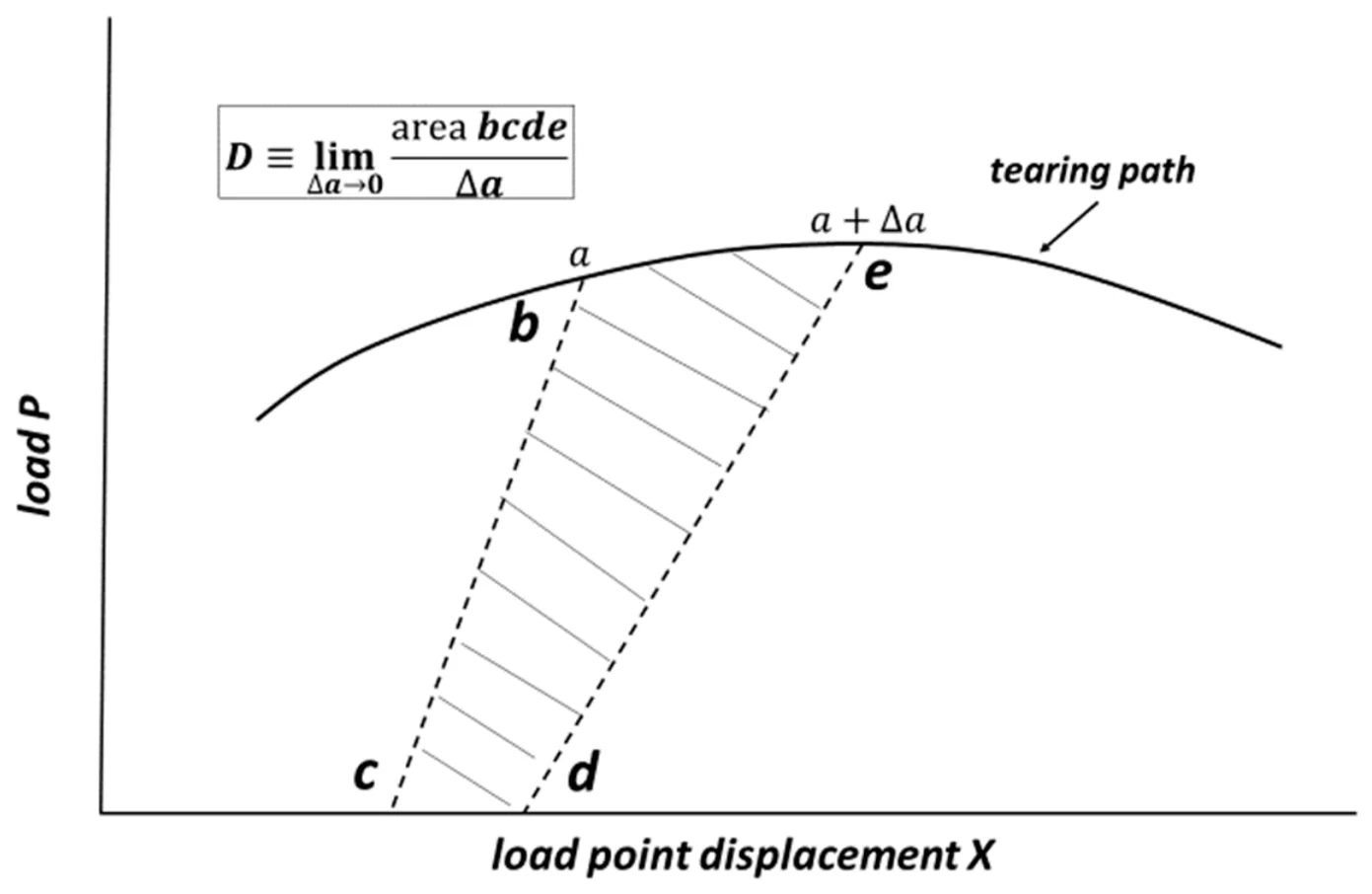
Graphical definition of the energy dissipation rate $D\left(a\right)$, where the elastic unloading paths may be either linear or non-linear. The formula at the top gives a numerical recipe for measuring the derivative from the shaded area under the tearing curve. Please note that the above graphical definition of D applies even in the case of non-linear elastic unloading curves.

**Figure 6:**
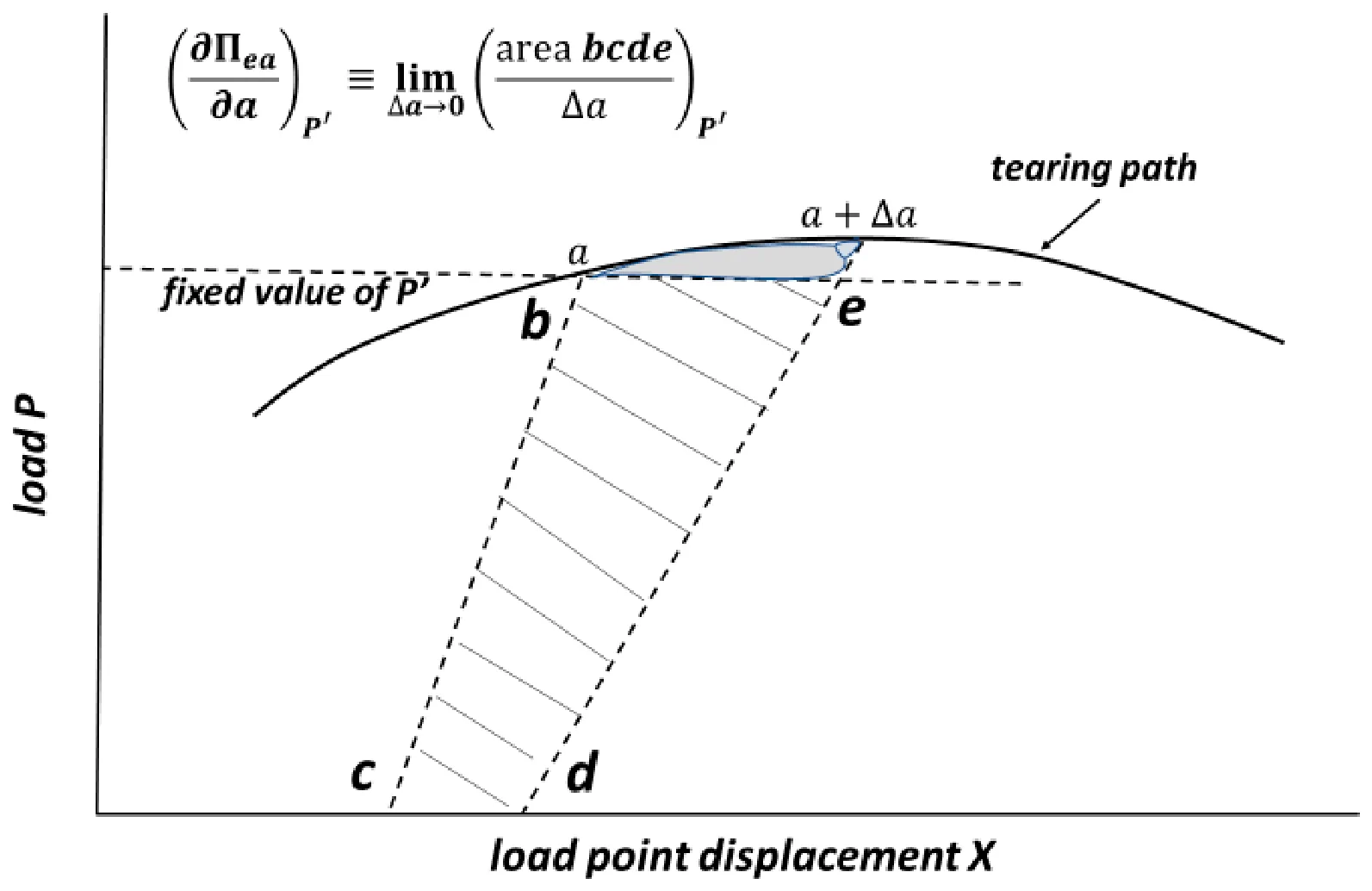
Graphical illustration of the fixed-load driving force ${\left(\frac{\partial \Pi_{ea}}{\partial a}\right)}_{P}$ at imminent fracture. Please note that the shaded area for ${\left(\frac{\partial \Pi_{ea}}{\partial a}\right)}_{P}$ in Figure 6 differs from that in the graphical definition of D in Figure 5 only by the small area painted in gray in Figure 6. This excess area, exhibiting second order variation in ∆P∆X, vanishes faster with decreasing ∆a than area bcde which exhibits first order variation in $\left\|\vec{cb}\right\|\cdot ∆X$. In the limit ∆a→0, the gray area in Figure 6 effectively vanishes, rendering the shaded areas in Figure 5 and Figure 6 equivalent, showing that ${\left(\frac{\partial \Pi_{ea}}{\partial a}\right)}_{P}=D$ at imminent fracture. Please note that this analysis applies regardless of whether the elastic unloading curves in Figure 5 and Figure 6 are linear or non-linear.

**Figure 7:**
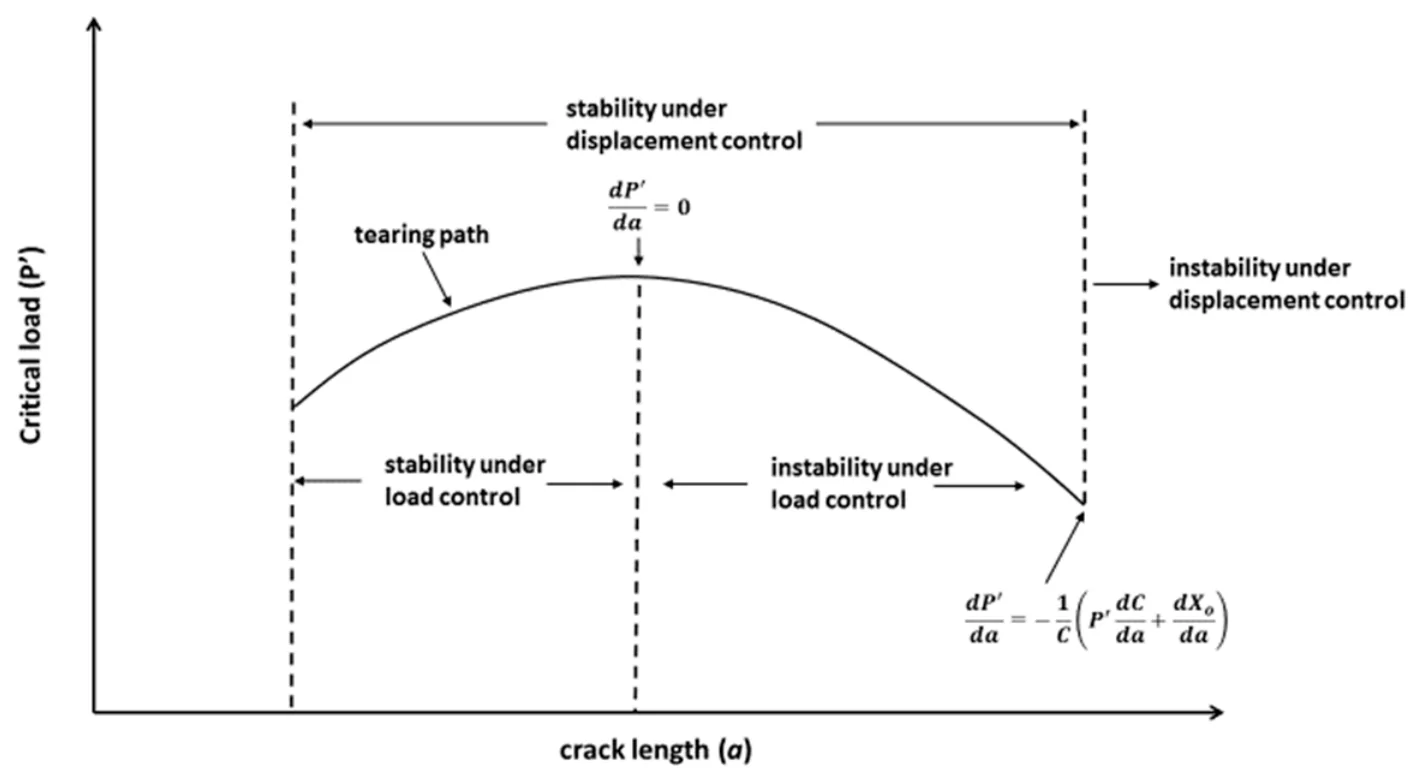
Predicted stability behavior of fracture-weakening and fracture-toughening systems under displacement and load-controlled conditions.

**Figure 8:**
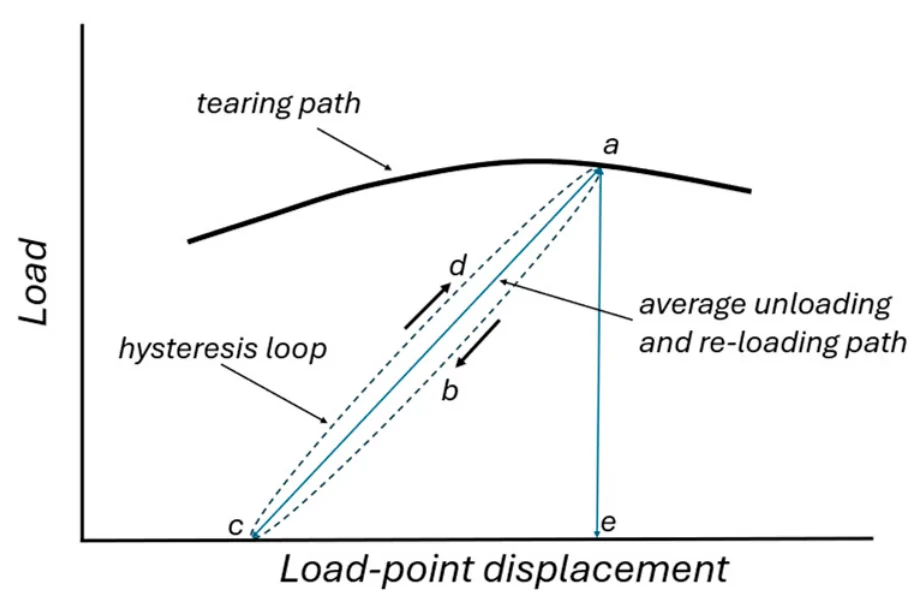
Illustration of hysteresis loop in load–displacement diagram.

**Figure A1:**
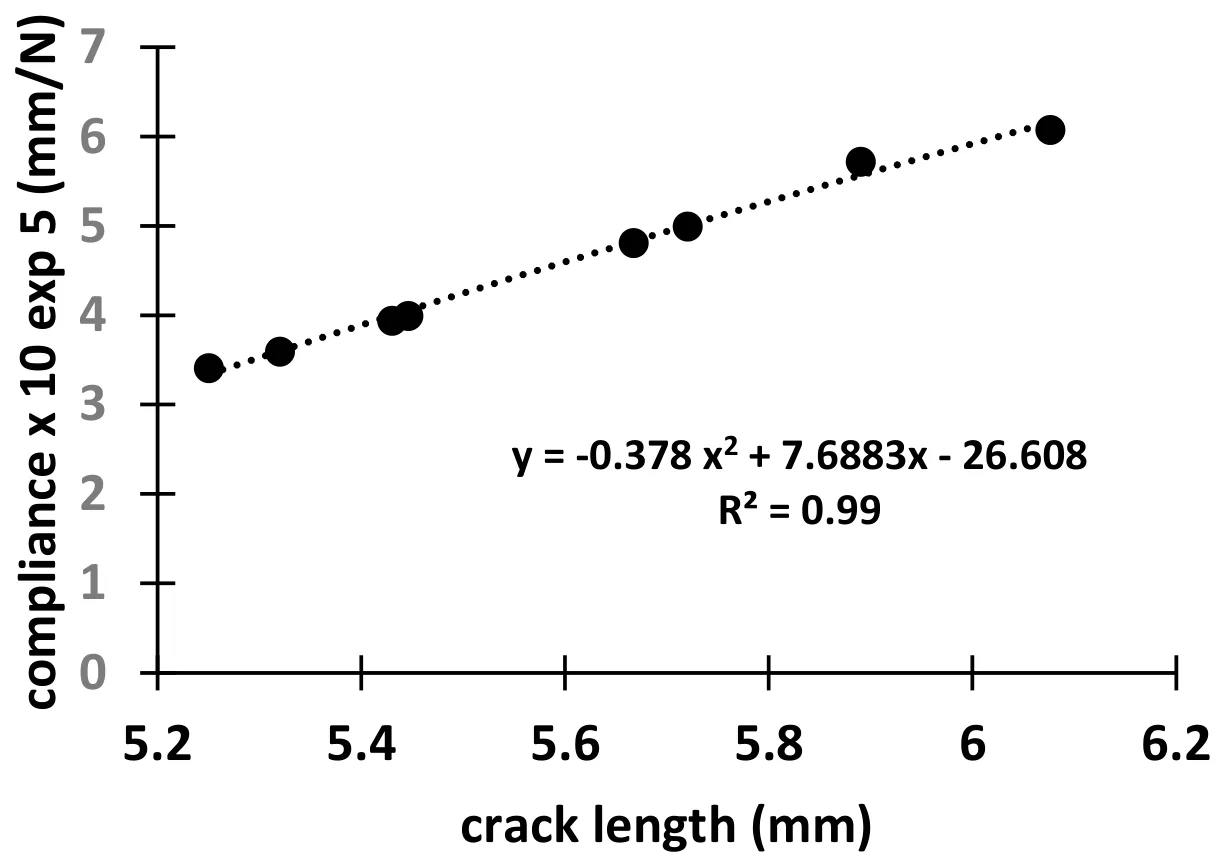
Compliance C as a function of crack length.

**Figure A2:**
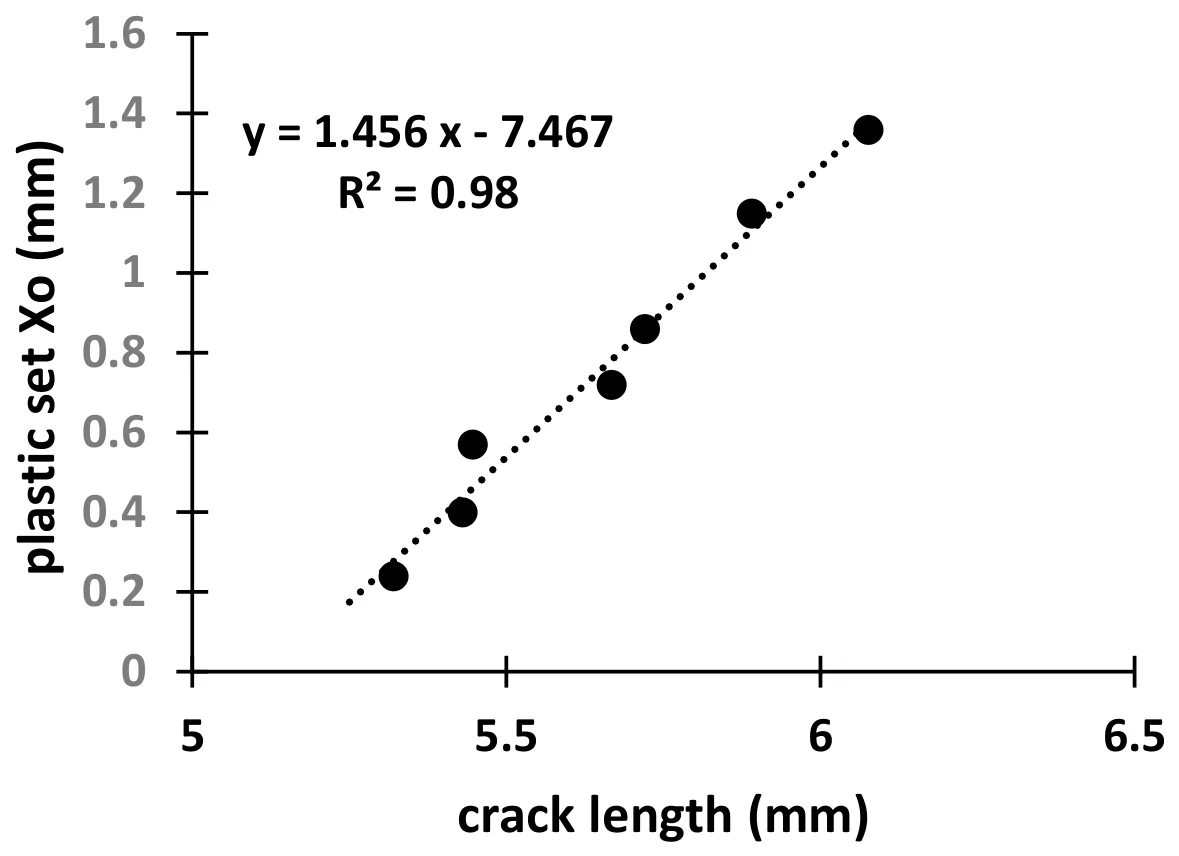
Plastic set X_o_ as a function of crack length.

**Figure A3:**
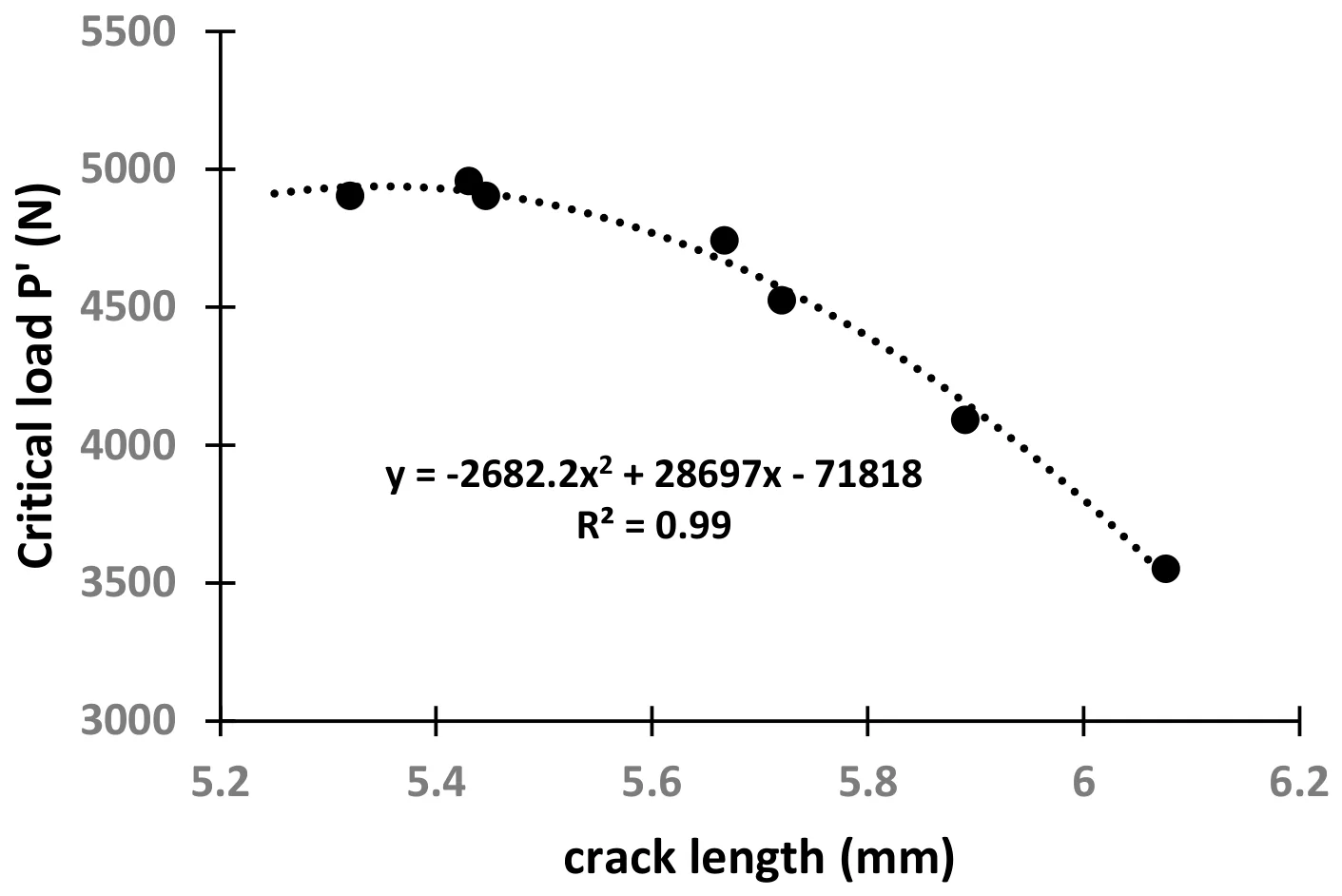
Critical load P’ as a function of crack length.

**Figure A4:**
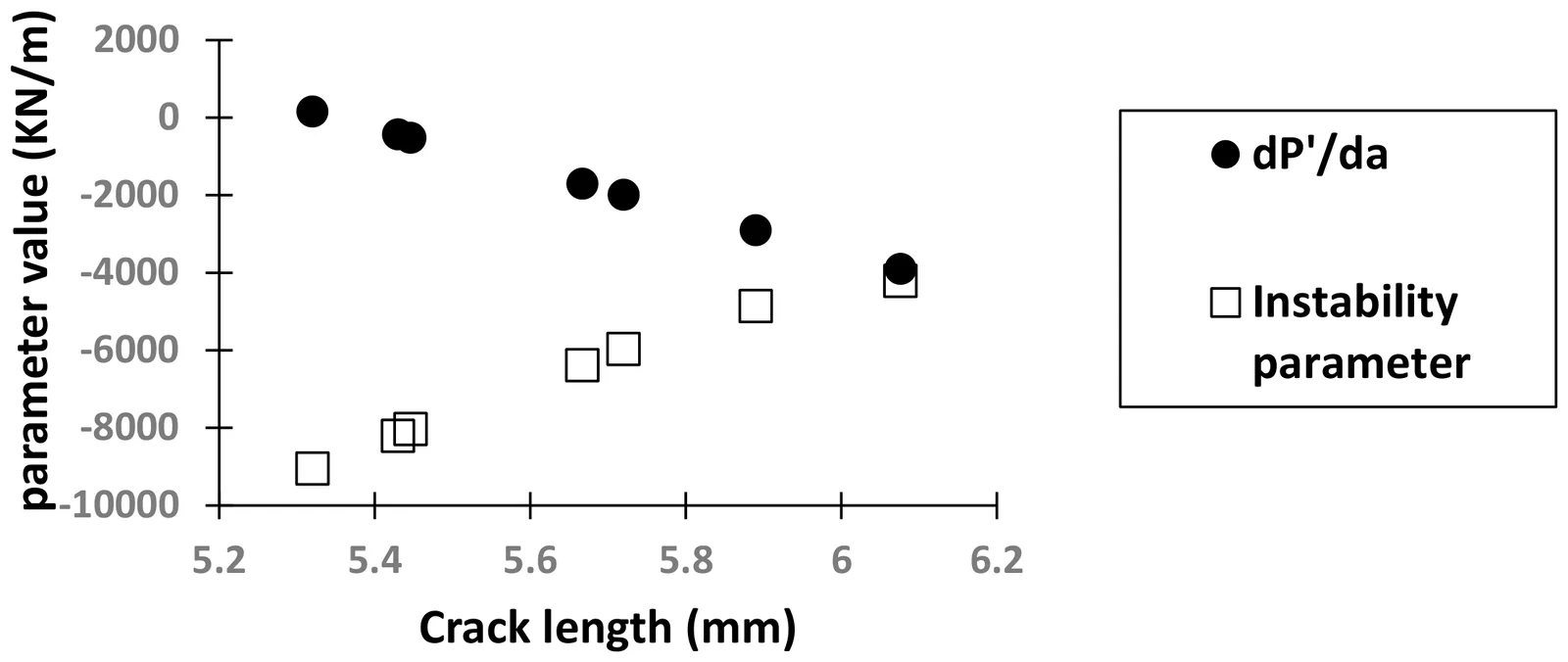
Evolution of slope $\frac{dP^{\prime }}{da}$ of the tearing curve in relation to the instability parameter $-\frac{1}{C}\left(P^{\prime }\frac{dC}{da}+\frac{dX_{o}}{da}\right)$ for a growing crack in a C(T) specimen of carbon steel under displacement-controlled loading conditions.

## Data Availability

The original contributions presented in this study are included in the article. Further inquiries can be directed to the corresponding author.

## Appendix A. Application of Instability Criteria to Published Experimental Data

### Appendix A.1. Basic Data

To illustrate use of the fixed grips instability criterion (53), we apply it to data from a fracture test on a C(T) specimen of carbon steel, published in Chapter 4 of a book by Saxena [10]. The objective of the book chapter was to illustrate methodology for determining J_R_ curves. Therefore, the data and associated calculations were presented in considerable detail, facilitating the present analysis.

Table A1 lists basic parameter values taken directly from the publication. Table A2 gives additional parameters calculated from the data in Table A1 and from a load–displacement diagram presented in the chapter. The plastic set parameters X_o_ in Table A2 were determined by extrapolating the linear elastic unloading curves in the load–displacement diagram to the X-axis. The derivatives dC/da, dX_o_/d*a* and dP’/d*a* in Table A2 were calculated by plotting C, X_o_ and P’ as functions of crack length (Figure A1, Figure A2 and Figure A3), and taking derivatives of the corresponding least-squares regression equations. These parameters were then substituted into relations (22) and (53) to determine the energy dissipation rate D and fixed-grips instability parameter $-\frac{1}{C}\left(P^{\prime }\frac{dC}{da}+\frac{dX_{o}}{da}\right)$, respectively, which are listed in the last two columns of Table A2.

**Table A1:** Fracture data for of a C(T) specimen of carbon steel, taken directly from Chapter 4 in Saxena [10].

Critical Load P’(N)	Crack Size *a* (mm)	Compliance C (mm/N)	K (MPa)$\sqrt{m}$	J $\left(KJ/m^{2}\right)$
0	5.25	3.41 × 10^−5^	------	------
4905	5.32	3.597 × 10^−5^	59.1	40.47
4959	5.43	3.94 × 10^−5^	61.1	61.11
4905	5.446	3.995 × 10^−5^	60.65	80.97
4742.8	5.667	4.812 × 10^−5^	61.6	100.62
4526.5	5.72	4.995 × 10^−5^	59.5	122.7
4093.9	5.89	5.722 × 10^−5^	56.3	148.1
3553.2	6.076	6.703 × 10^−5^	51.5	174.45

**Table A2:** Parameters calculated from data in Table A1 and a load–displacement diagram provided in [10].

Crack Size *a* (mm)	Plastic Set X_o_ (mm)	$\frac{dC}{da}$ (N^−1^)	$\frac{dX_{o}}{da}$	$\frac{dP^{\prime }}{da}$ (KN/m)	D $\left(KJ/m^{2}\right)$	Instability Parameter $-\frac{1}{C}\left(P^{\prime }\frac{dC}{da}+\frac{dX_{o}}{da}\right)$(KN/m)
5.25	0	---------			------	
5.32	0.24	3.667 × 10^−5^	1.456	158.4	75.81	−9047.8
5.43	0.4	3.584 × 10^−5^	1.456	−431.7	76.59	−8205.7
5.446	0.57	3.572 × 10^−5^	1.456	−517.5	75.69	−8029.5
5.667	0.72	3.405 × 10^−5^	1.456	−1703.1	72.87	−6381.3
5.72	0.86	3.651 × 10^−5^	1.456	−1987.4	69.33	−5963.3
5.89	1.15	3.237 × 10^−5^	1.456	−2899.3	62.29	−4859.1
6.076	1.36	3.096 × 10^−5^	1.456	−3897.1	53.87	−4206.1

### Appendix A.2. Results

#### Appendix A.2.1. General Observations on Fracture Parameters

The threefundamental fracture-related parameters listed in Table A1 and Table A2 are J, K and D. In elastic–plastic J-integral theory, the parameter K arises more or less as a formality, as part of the parameter $\frac{K^{2}}{E}$ used to calculate the so-called “elastic” component of the J-integral. However, in the thermodynamic analysis of Section 3, K arises at a more fundamental level as an excess elastic stress intensity parameter related to D and the thermodynamic driving forces governing fracture. It is perhaps significant in Table A1 and Table A2 that experimental values of K and D remained relatively invariant during crack extension, never deviating by more than 15 and 30 percent from the corresponding initial values, whereas the elastic–plastic J integral changed by more than 400 percent relative to the initial value.

#### Appendix A.2.2. Stability Analysis

Figure A4 compares the evolution of the tearing curve slope $\frac{dP^{\prime }}{da}$ and the fixed-grips instability parameter $-\frac{1}{C}\left(P^{\prime }\frac{dC}{da}+\frac{dX_{o}}{da}\right)$ as functions of crack extension. The slope $\frac{dP^{\prime }}{da}$ was negative over most of the loading trajectory, indicating fracture weakening conditions. Nevertheless, loading was always stable, as evidenced by the empirical fact that loads were quasi-statically sustainable throughout the course of the experiment. Consistent with this fact, Figure A4 shows that the fracture toughening parameter $\frac{dP^{\prime }}{da}$ was always more positive than the instability parameter $-\frac{1}{C}\left(P^{\prime }\frac{dC}{da}+\frac{dX_{o}}{da}\right)$, as predicted by criterion (53) for stable displacement-controlled behavior. As crack growth increased, however, the fracture toughening parameter $\frac{dP^{\prime }}{da}$ became increasingly negative and the instability parameter $-\frac{1}{C}\left(P^{\prime }\frac{dC}{da}+\frac{dX_{o}}{da}\right)$ became more positive, so that the two parameters monotonically approached each other as crack growth proceeded. At the point where the experiment was terminated (presumably because stable loading could no longer be sustained) the two curves appeared to very nearly cross over each other, again as predicted by criterion (53) for onset of instability. Unfortunately, since Saxena’s book chapter does not indicate at what point instability actually occurred in the experiment, there is an element of speculation in assuming that termination of the experiment coincided with the onset of instability. Nevertheless, the fact that the predicted condition for instability was monotonically and very nearly approached toward the end of the experiment provides reasonable evidence for validity of the criterion, at least in this particular case. As noted previously, full validation of the criterion will require testing it over a wide range in loading configurations, using materials with variable degrees of plasticity and fracture toughening/softening behaviors.
